# Transcriptomic correlates of cell cycle checkpoints with distinct prognosis, molecular characteristics, immunological regulation, and therapeutic response in colorectal adenocarcinoma

**DOI:** 10.3389/fimmu.2023.1291859

**Published:** 2023-12-08

**Authors:** Heng Wang, Wei Wang, Zhen Wang, Xu Li

**Affiliations:** ^1^ Department of Colorectal Surgery, Shanghai Yangpu Hospital of Traditional Chinese Medicine, Shanghai, China; ^2^ Department of Colorectal Surgery, The First Affiliated Hospital of Naval Medical University, Shanghai, China

**Keywords:** colorectal adenocarcinoma, cell cycle checkpoint, CCCs signature, overall survival, molecular characteristics, immunological regulation, therapeutic response, GDSC

## Abstract

**Backgrounds:**

Colorectal adenocarcinoma (COAD), accounting for the most common subtype of colorectal cancer (CRC), is a kind of malignant digestive tumor. Some cell cycle checkpoints (CCCs) have been found to contribute to CRC progression, whereas the functional roles of a lot of CCCs, especially the integrated role of checkpoint mechanism in the cell cycle, remain unclear.

**Materials and methods:**

The Genomic Data Commons (GDC) The Cancer Genome Atlas (TCGA) COAD cohort was retrieved as the training dataset, and GSE24551 and GSE29623 were downloaded from Gene Expression Omnibus (GEO) as the validation datasets. A total of 209 CCC-related genes were derived from the Gene Ontology Consortium and were subsequently enrolled in the univariate, multivariate, and least absolute shrinkage and selection operator (LASSO) Cox regression analyses, finally defining a CCC signature. Cell proliferation and Transwell assay analyses were utilized to evaluate the functional roles of signature-related CCCs. The underlying CCC signature, molecular characteristics, immune-related features, and therapeutic response were finally estimated. The Genomics of Drug Sensitivity in Cancer (GDSC) database was employed for the evaluation of chemotherapeutic responses.

**Results:**

The aberrant gene expression of CCCs greatly contributed to COAD development and progression. Univariate Cox regression analysis identified 27 CCC-related genes significantly affecting the overall survival (OS) of COAD patients; subsequently, LASSO analysis determined a novel CCC signature. Noticeably, *CDK5RAP2*, *MAD1L1*, *NBN*, *RGCC*, and *ZNF207* were first identified to be correlated with the prognosis of COAD, and it was proven that all of them were significantly correlated with the proliferation and invasion of HCT116 and SW480 cells. In TCGA COAD cohort, CCC signature robustly stratified COAD patients into high and low CCC score groups (median OS: 57.24 months vs. unreached, *p*< 0.0001), simultaneously, with the good AUC values for OS prediction at 1, 2, and 3 years were 0.74, 0.78, and 0.77. Furthermore, the prognostic capacity of the CCC signature was verified in the GSE24551 and GSE29623 datasets, and the CCC signature was independent of clinical features. Moreover, a higher CCC score always indicated worse OS, regardless of clinical features, histological subtypes, or molecular subgroups. Intriguingly, functional enrichment analysis confirmed the CCC score was markedly associated with extracellular, matrix and immune (chemokine)-related signaling, cell cycle-related signaling, and metabolisms. Impressively, a higher CCC score was positively correlated with a majority of chemokines, receptors, immunostimulators, and anticancer immunity, indicating a relatively immune-promoting microenvironment. In addition, GSE173839, GSE25066, GSE41998, and GSE194040 dataset analyses of the underlying CCC signature suggested that durvalumab with olaparib and paclitaxel, taxane-anthracycline chemotherapy, neoadjuvant cyclophosphamide/doxorubicin with ixabepilone or paclitaxel, and immunotherapeutic strategies might be suitable for COAD patients with higher CCC score. Eventually, the GDSC database analysis showed that lower CCC scores were likely to be more sensitive to 5-fluorouracil, bosutinib, gemcitabine, gefitinib, methotrexate, mitomycin C, and temozolomide, while patients with higher CCC score seemed to have a higher level of sensitivity to bortezomib and elesclomol.

**Conclusion:**

The novel CCC signature exhibited a good ability for prognosis prediction for COAD patients, and the CCC score was found to be highly correlated with molecular features, immune-related characteristics, and therapeutic responses, which would greatly promote clinical management and precision medicine for COAD.

## Introduction

Colorectal cancer (CRC), one of the most common digestive malignancies, is still contributing a heavy burden to human health with a high mortality rate, with almost a million new cases annually all over the world (1). Colorectal adenocarcinoma (COAD) is the most prevalent subtype of CRC, and most patients are diagnosed in advanced stages. In the past decades, colonoscopy, flexible sigmoidoscopy, and computed tomography colonography have improved CRC screening and prevention (2). Meanwhile, the classification of tumor node metastasis stage (T, N, M stage) and clinical stage (I, II, III, IV stage) established by the Union for International Cancer Control and American Joint Commission on Cancer (UICC and AJCC) provided guidelines for the selection of the therapeutic regimens in CRC (3, 4). However, within CRC patients with the same clinical stage, their prognosis was very distinct, which caused tremendous difficulties in CRC management (5). Classic criteria, such as TNM and clinical staging system, have limitations in CRC management. Currently, we need to develop more robust risk stratification tools to guide clinicians to tailor treatment interventions.

With the rapid development of next-generation sequencing (NGS), comprehensive genomic profiling of CRC has revealed that genetic (*KRAS*, *TP53*, *PIK3CA*, and *APC*) and epigenetic (hypomethylated *CEP55*, *FOXD3*, *FOXF2*, *GNAO1*, *GRIA4*, and *KCNA5*) alterations potentially contribute to the colorectal carcinogenesis (6, 7). However, the genomic landscape is insufficient to demonstrate the suffused changes in gene expressions and alterations of cellular function (8, 9). In tumor tissues, genetic and epigenetic alterations could directly cause the disturbed gene expression, possibly resulting in tumor cell invasiveness. Integrated omics studies confirmed that gene regulatory networks or protein–protein interaction networks representing biological processes have a better ability to predict CRC tumorigenesis and progression (10) than a single gene or protein. Quantified risk scores based on multigene expression have become promising diagnostic and prognostic biomarkers with great clinical value, which have significantly high accuracy and efficiency in predicting the diagnosis and prognosis of CRC patients, even response to therapeutics and risk of recurrence, etc. The mRNA-based signatures, such as Veridex (11), OncotypeDX (12), Coloprint (13), and GeneFx Colon (14) demonstrate their applicability in predicting survival outcomes of CRC patients, and these prognostic models have been validated in a separate study (15). Furthermore, the consensus molecular subtypes (CMSs: CMS1, CMS2, CMS3, and CMS4) classifier based on comprehensive transcriptional profiling could divide CRC patients into four CMSs with distinguishing clinical features (16), and this model has been shown to be extremely effective in predicting therapeutic responses that help clinical decision making in CRC (17). While the stroma plays an important role in patient stratification and prognosis, the accuracy of prognosis prediction via CMS is negatively affected due to stromal-derived intratumor heterogeneity (18). Compared to the stromal-dependent model CMS, CRC intrinsic gene expression signatures avoid misclassification of CRC patients (19). However, these biomarkers are predominantly based on TNM and/or microsatellite instability (MSI) stratification systems, gradually reducing their values to risk evaluation in clinical practices (20). Consequently, novel and robust predictive biomarkers are urgently needed to assist clinicians in better managing CRC patients and proposing personalized medicine.

Cancer cells, a group of cells dividing continuously and excessively, undergo aberrant cell cycle progression. Recent works have shown strong evidence that the disruption of cell cycle control plays an irreplaceable role in continuous cell division (21). During different cell cycle phases in eukaryotic cells, checkpoints, including DNA damage checkpoints, DNA replication stress checkpoints, and mitotic checkpoints, function to prevent DNA damage and chromosome missegregation (22). In brief, cell cycle checkpoints (CCCs) are responsible for inspecting the cell cycle to ensure the quality of DNA replication and chromosome distribution. For example, defects in checkpoint regulatory components, such as ataxia telangiectasia mutated (*ATM*), cause decreased efficiency of DNA repair and increased genomic instability widely in tumors (23). DNA damage checkpoint kinase CHK1 regulates DNA replication, phase transition, and mitosis, and the elevated expression of *CHK1* is found to be significantly associated with prognosis, recurrence, and even therapy resistance in acute myeloid leukemia, hepatocellular carcinoma, breast carcinoma, and colorectal carcinoma (24). The dysregulated spindle checkpoint gene *MAD2* is involved in colorectal carcinogenesis, and the overexpressed level of MAD2 protein is more frequently observed in CRC patients with lymph node metastasis (25, 26). Moreover, the overexpression of spindle assembly checkpoints, including *BUBR1*, *MAD1*, *MAD2*, *MPS1*, and *CDC20*, has been reported in several cancer types. While the overexpression of *MAD2* can lead to the hyperactivation of checkpoint (27), the expression of *MAD1* has the opposite effect (28). Although the role of some cell cycle checkpoints in cancer progression has been described in detail, current studies focus on single checkpoints. Nevertheless, the prognostic significance of integrative transcriptional characterization of CCCs in CRC remains elusive. Moreover, the functional role of a lot of CCCs in CRC remains to be further investigated.

In the present study, comprehensive gene expression profiles of COAD patients with clinical information were derived from TCGA, which was used to explore the association between the expression of checkpoint mechanisms in the cell cycle and the OS of COAD patients. The Least absolute shrinkage and selection operator (LASSO) Cox regression analysis was conducted to develop a CCC gene-based signature and a related CCC score system, of which the prognostic ability was estimated by the AUC value and further verified in additional independent datasets from the GEO database. Subsequently, the molecular characteristics and tumor microenvironmental status were further investigated to disclose the distinct molecular mechanisms and immunological regulation associated with the CCC signature. Ultimately, the CCC signature was employed to predict the (chemo)therapeutic response, including chemotherapy, neoadjuvant therapy, frontline chemotherapy, immunotherapy, etc. The findings in the present study provided deep insights into the functional roles of cell cycle checkpoints affecting the prognosis, molecular mechanisms, and immunological regulation of COAD, which would help accelerate the development of precision medicine.

## Materials and methods

### Data acquisition and processing

The gene expression profiles of 359 COAD patients with clinical data were retrieved from the GDC TCGA COAD cohort from the website of UCSC xena (https://xenabrowser.net/datapages/), which was regarded as the training dataset. Clinical features, such as age, gender, clinical stages, TNM stages, and others, were all included. The GSE24551 (containing 160 COAD patients, no patient in clinical stage IV (29),) and GSE29623 (containing 65 COAD patients (30),) datasets were downloaded from GEO (https://www.ncbi.nlm.nih.gov/geo/) as the validation datasets. GSE153412 (31), GSE196263 (32), GSE173839 (33), GSE25066 (34–36), GSE41998 (37), GSE194040 (38), and GSE48276 (39) datasets were retrieved to explore the correlation between the transcriptomic profile of CCCs and therapeutic response.

A total of 209 CCC-related genes involved in the checkpoint mechanisms were acquired from the Gene Ontology Consortium (http://geneontology.org/) (40). Heatmap was performed via using the package “pheatmap” to visualize differentially expressed genes (DEGs, |log_2_ fold change (FC)|>1, adjust *p*-value< 0.05) between COAD and normal samples, namely GDC TCGA COAD from the website of UCSC xena (https://xenabrowser.net/datapages/). The DEG analysis was conducted using the “DeSeq2” package.

### Development, evaluation, and validation of the CCC signature

The CCC-derived genes were analyzed via univariate Cox regression analysis. The LASSO Cox regression analysis was then utilized with the package “glmnet”, and the optimal penalty regularization index λ was selected via the 10-fold crossvalidation to prevent the overfitting effects. Eventually, OS-related CCCs were determined to construct the risk signature, and the CCC score (based on the CCC signature) of each COAD patient was calculated as the following formula:


$$\text{CCC score }=\;\sum_{j=1}^{n}{\text{Expression}}_{j}{*\text{Coefficient}}_{j}$$

Where *n* is the gene number in the CCC signature, Expression*_j_* is the expression level of gene *j*, and Coefficient*_j_* is the coefficient value of gene *j* generated via multivariate Cox regression analysis. The COAD patients from TCGA cohort were divided into high and low CCC score groups according to the median CCC score, as the cutoff value.

The Kaplan–Meier (KM) curve analysis, by using the packages “survival” and “survminer”, was conducted to compare the difference in patients’ OS between two subgroups in training and validation cohorts. Moreover, the receiver operating characteristic (ROC) curve was performed by using the package “timeROC”, and the value of area under ROC (AUC) was analyzed to evaluate the performance of CCC signature in prognosis prediction. Additionally, the correlation analysis was used to assess the relationship between CCC signature and clinical features.

Subsequently, the prognostic significance of the CCC signature was further verified in two independent validation datasets. The KM curve, the value of the AUC, and Cox regression analyses were also conducted in these additional COAD cohorts. The multivariate Cox proportional hazards regression model was utilized to confirm the independence of CCC signature and clinical features, including diagnosis age, gender, TNM stage, and clinical stage, by using the packages “rms” and “survminer”.

### Immunohistochemistry analysis of CCC-related protein expression

The immunohistochemistry (IHC) data, obtained by using the COAD tissue macroarray staining from the Human Protein Atlas (HPA, https://www.proteinatlas.org/), were downloaded to investigate the cellular distribution of the CCC signature-related gene expression at the protein level in human COAD samples. In the present study, a total of eight to 12 COAD samples were enrolled in the IHC staining analysis of each CCC signature-related gene protein expression. Regarding the CCC signature, the IHC staining information of BRCA1 (HPA034966), BRSK1 (HPA061719), CCNB1 (CAB003804), CDC25C (CAB003800), CDK5RAP2 (HPA046529), CDKN1B (CAB021888), CLOCK (HPA001867), CNOT6 (HPA044568), CNOT6L (HPA042688), MAD1L1 (CAB015338), MAD2L1 (HPA003348), NBN (HPA001429), ORC1 (HPA027450), PLK1 (HPA053229), RGCC (HPA035638), ZNF207 (HPA017013), and ZW10 (HPA055410) was available in the HPA database, whereas no information could be retrieved for BUB1, CNOT7, and SETMAR protein expression. The IHC staining data were evaluated by experts in the HPA, who eventually determined whether the results of IHC staining or not detected.

### RNA interference and overexpression system construction

The CCC signature-related gene silence in the HCT116 and SW480 cell lines was performed by using siRNA technology. SiRNAs for targeted genes were obtained from Tsingke Biotech (Beijing, China). In accordance with the manufacturer’s instruction, transfections of siRNA into cells were performed via using Lipofectamine® 3000 reagent (Thermo Fisher, MA, USA) at a final concentration of 20 nM. The scramble siRNA was used in the control group, and the following siRNAs were used for RNA interference: siRNA#1 against *CDK5RAP2*: 5′-UGGAAGAUCUCCUAACUAA-3′; siRNA#2 against *CDK5RAP2*: 5′-CUAUGAGACUGCUCUAUCA-3′; siRNA#1 against *MAD1L1*: 5′-CAGCGATTGTGAAGAACAT-3′; siRNA#2 against *MAD1L1*: 5′-GCAGCATGACTGACAGACA-3′; siRNA#1 against *RGCC*: 5′-CTAAAGAGCTCGAAGACTT-3′; siRNA#2 against *RGCC*: 5′-CAATACTTCAGGGGCTTTGA-3′.

To construct a gene overexpression system, the pCMV plasmid was transfected using Lipofect8000 (Thermo Fisher, CA, USA) to induce gene overexpression. pCMV-*ZNF207*-GFP and pCMV-*NBN*-GFP vectors were obtained from COBIOER Inc. (Nanjing, China). cDNAs for human genes *ZNF207* and *NBN* were synthesized by Sino Biological Inc. (Beijing, China).

### qRT-PCR and Western blotting analysis

The silencing efficiency of siRNA was determined by qRT-PCR analysis. Total RNA was extracted from HCT116 and SW480 cells and then reversely transcribed into cDNA with a reverse transcription kit (Applied Biosystems Inc., OK, USA). Quantified PCR was performed by applying the SYBR Green Real-Time PCR Master Mixes (Thermo Fisher, CA, USA) on an ABI7500 instrument (Applied Biosystems Inc., CA, USA). GAPDH was used as an internal control. The following primers were used for qRT-PCR analysis: *CDK5RAP2* forward primer: 5′-GGCCCCACTGAACATATCTACA-3′; *CDK5RAP2* reverse primer: 5′-CACCTTCTTTCGAGCATCTTCTT-3′; *MAD1L1* forward primer: 5′-TGGACTGGATATTTCTACCTCGG-3′; *MAD1L1* reverse primer: 5′-CCTCACGCTCGTAGTTCCTG-3′; *RGCC* forward primer: 5′-CGCCACTTCCACTACGAGG-3′; *RGCC* reverse primer: 5′-CAGCAATGAAGGCTTCTAGCTC-3′.

The gene *ZNF207*- and *NBN*-overexpressed cell lines were testified by Western blotting analysis. The cells were lysed by using RIPA (50 mM Tris, 150 mM NaCl, 1% NPNP-40, 1% sodium deoxycholate, 0.05% SDS, pH = 7.4). The quantification of proteins was performed by using the BCA Kit (Bio-Rad Inc., CA, USA). Subsequently, SDS-polyacrylamide gel electrophoresis was performed and then transferred onto polyvinylidene difluoride, followed by blocking with 5% BSA, and incubated overnight at 4° with the first antibody (anti-GFP, 1:5,000). The next day, protein samples were incubated with a secondary antibody for 1 h, followed by visualization.

### Cell proliferation and Transwell assay

HCT116 or SW480 cells were pretreated with siRNA or vectors. Cell proliferation level was estimated by using the Cell Counting Kit-8, namely CCK-8 assay (Beyotime, Shanghai, China), according to the manufacturer’s instructions. Subsequently, cell viability was performed using a microplate reader at 24, 48, and 72 h with the absorbance at 450 nm. In this experiment, HCT116 and SW480 cells were analyzed and repeated independently three times. A Transwell assay was conducted to evaluate the invasion rate of HCT116 and SW480 cells; 5 × 10^4^ cells were seeded in a Transwell chamber (8 μm, Thermo Fisher, CA, USA) containing 300 μL of culture medium (10% FBS). Subsequently, 1 mL of FBS-free culture medium was added to the lower chamber (a 24-well plate); 24 h later, the chamber was fixed with paraformaldehyde and stained with crystal violet. Cells were visualized, and cell numbers were counted under the microscope (Leica, Wetzlar, Germany).

### Exploration of CCC signature in distinct molecular subtypes

Compared to wild types, it was generally believed that *KRAS*, *NRAS*, or *BRAF* (*KRAS*/*NARS*/*BRAF*) had a great influence on prognosis in CRC patients with mutated *APC* or *TP53* (41). Therefore, according to the status of these genes, in this study, COAD patients could be assigned to the various mutant subtypes. Additionally, the MSI (highly associated with deficient mismatch repair) stratification system, defined by the MSI sensor score in this study, could divide COAD patients into MSI-H (MSI sensor score ≥ 10), MSI-L, and MSS (MSI sensor score< 10) subgroups. In addition, as described before, CMS subtyping is now widely accepted and reliable for CRC. The CMS classifier was defined and calculated with reference to the previously reported (16).

### Molecular characteristics associated with CCC signature

The genomic alteration profiles for each COAD patient from the TCGA cohort, shown in the oncoprint plots, between high and low CCC score groups were investigated by using the package “maftools” to illustrate a variety of alteration events (substitution, frameshift, multi-hit, etc.) of the top 20 prevalently altered genes. The Kyoto Encyclopedia of Genes and Genomes (KEGG, www.kegg.jp/ (42),) pathway enrichment analysis was conducted using the “clusterProfiler” package, and the significantly enriched signaling pathways were annotated in KEGG and visualized by utilizing the “ggplot2” package. HALLMARK gene set enrichment analysis (GSEA), based on GSEA v.3.0, was also performed to explore the enriched pathways correlated with the CCC signature, and the enrichment score (ES) would be further normalized for each involved gene set.

### Immune characteristics and anti-cancer immunity analysis

To explore the tumor microenvironment (TME) associated with CCC score, the previously described ESTIMATE algorithm (43) was employed to assess the stromal and immune microenvironments, and CIBERSORT algorithm analysis (44) deconvoluting the expression profile (containing 547 genes) of immune-related cells was conducted to infer the difference in the proportion of 22 tumor-infiltrated lymphocytes between high and low CCC score groups, respectively. Furthermore, several indexes, including immunomodulator gene expression, tumor immune cell infiltration, cancer immunity cycle, and inhibitory immune checkpoint-related gene expression, were employed for the evaluation of the immune-related features, anticancer immunity, and microenvironmental status in COAD. A total of 122 immunomodulators (chemokines, paired receptors, MHC molecules, and immunostimulators), which had been reported previously, were collected to estimate the immunomodulation of TME (45). The cancer immunity cycle was reviewed to represent anticancer immunity, and it contained seven steps: step 1: release of cancer cell antigens; step 2: cancer antigen presentation; step 3: priming and activation; step 4: trafficking of immune cells to cancer cells; step 5: immune cell infiltration into the tumor; step 6: recognition of cancer cells by T cell; and step 7: killing of cancer cell (46). The activities of these steps were performed using a single sample gene set enrichment analysis (ssGSEA), based on the transcriptomic data of each patient sample (47). To decrease the errors, more algorithms were performed to assess the infiltrating levels of tumor immune cells (mainly CD8+, macrophage, dendritic cells, natural killer (NK) cells, and Th1 cells), including the TIMER, CIBERSORT, QUANTISEQ, MCPCOUNTER, XCELL, and EPIC algorithms. The profile of inhibitory immune checkpoints was obtained from the study of Auslander (48).

### Evaluation of therapeutic response underlying CCC signature

Initially, the GSE153412 dataset about the therapeutic response evaluation of COAD cancer cell lines (HCT116 and HT29) and the GSE196263 dataset of CMS4 subtype colon cancer patients receiving imatinib treatment were downloaded to explore the potential predictive role of CCC score in the therapeutic response for COAD patients. Moreover, the GSE173839 (HER2-negative stage II/III breast cancer patients on the durvalumab with olaparib and paclitaxel arm: 29 responders vs. 42 nonresponders), GSE25066 (breast cancer patients treated with taxane-anthracycline chemotherapy: 99 patients with pathological complete response (pCR) vs. 389 patients with residual disease (RD)), and GSE41998 (early-stage breast cancer patients receiving neoadjuvant cyclophosphamide/doxorubicin with ixabepilone or paclitaxel: 69 responders vs. 184 responders) datasets were also downloaded to further evaluate the predictive ability of the CCC signature in the therapeutic response. The dataset GSE194040 (the I-SPY2-990 mRNA/RPPA Data Resource) contained gene expression and clinical data for 987 patients from 10 arms of the neoadjuvant I-SPY2 TRIAL for aggressive early-stage breast cancer. The GSE48276 dataset included 128 muscle-invasive bladder cancer patients receiving frontline chemotherapy. In addition, the Genomics of Drug Sensitivity in Cancer (GDSC, https://www.cancerrxgene.org/) database was usually employed to predict chemotherapeutic response; therefore, the GDSC database was further utilized by the “pRRophetic” package. The value of half maximal inhibitory concentration (IC50) was used to assess the chemotherapeutic response.

### Statistical analysis

The statistical data analyzed in this study were all performed in R Studio (https://rstudio.com/). The univariate and multivariate analyses investigating the prognosis-related risk factor(s) were conducted via the Cox proportional hazards regression model. The exploration of the survival difference of OS via the KM curves was based on a log-rank test. The Chi-square test, Fisher’s test, Wilcoxon-rank test, and Kruskal–Wallis *H* test were utilized to conduct the correlation analysis between the CCC signature and clinical features. The (adjusted) *p*-value< 0.05 was considered statistically significant.

## Results

### Dysregulation of CCCs in the development and progression of COAD

**Figure 1:**
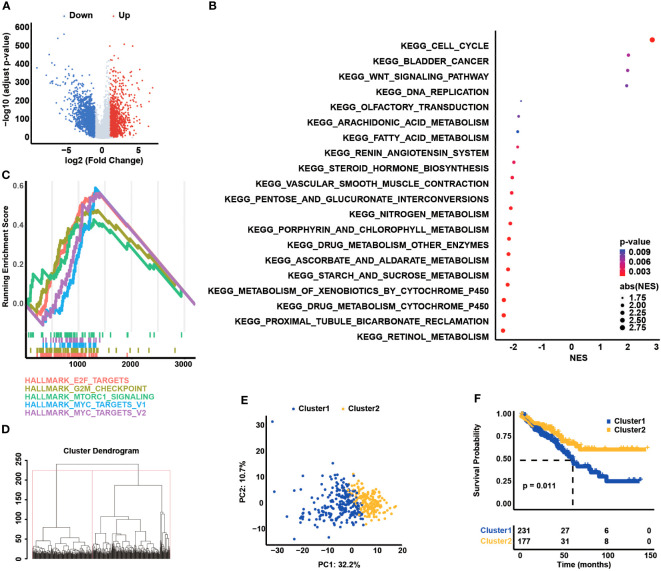
Dysregulation of cell cycle checkpoints (CCCs) in the development and progression of colorectal carcinoma (COAD). **(A)** The differentially expressed gene (DEG) analysis between normal and tumor tissues. **(B)** The functional enrichment analysis is based on the DEGs between normal and tumor tissues. **(C)** The gene set enrichment analysis is the foundation of the HALLMARK pathways. **(D)** The unsupervised hierarchy clustering analysis is based on the transcriptomic profile of CCCs in the TCGA COAD cohort. **(E)** The principal component analysis (PCA) of two separated clusters. **(F)** The Kaplan–Meier curve analysis of two divided clusters.

Initially, the DEGs analysis between COAD and normal samples showed that there were 3,890 DEGs in total, including 1,871 upregulated and 2,019 downregulated genes in COAD samples, with a statistically significant difference (|log_2_ (FC)|>1, adjust *p*-value< 0.05, **Figure 1A**). The functional enrichment analysis (**Figures 1B, C**) suggested that those DEGs were mainly enriched in the signaling pathway of the cell cycle, illustrating that cell cycle-related dysregulation had a great influence on the development of COAD. Based on the expression profiling of 209 CCCs, COAD patients were divided into two clusters via the unsupervised hierarchy clustering analysis (**Figure 1D**). Moreover, the principal component analysis (PCA) confirmed that the expression profiling of CCCs apparently distinguished these two clusters (**Figure 1E**). Intriguingly, these two clusters had the distinct OS (median OS of cluster 1 vs. cluster 2: 61.84 months vs. unreached, *p* = 0.011, **Figure 1F**), suggesting that dysregulation of CCC expression was markedly associated with the prognosis of COAD patients.

### Establishment of CCC gene-based signature in COAD

**Figure 2:**
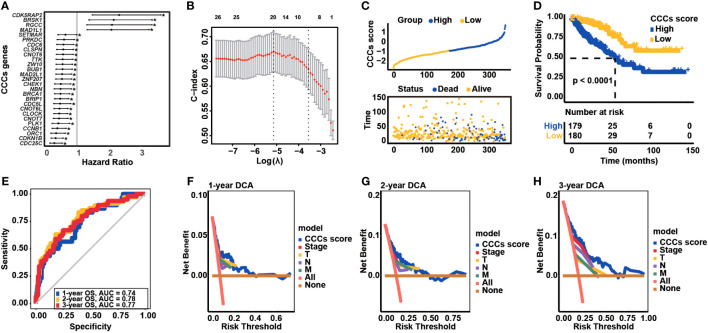
Transcriptomic correlates of cell cycle checkpoints (CCCs) with the overall survival (OS) of colorectal carcinoma (COAD) and construction of a novel CCC signature. **(A)** The univariate Cox regression analysis identified the OS-related CCCs in COAD. **(B)** The least absolute shrinkage and selection operator (LASSO) Cox regression analysis for the construction of a novel CCC signature. **(C)** COAD patients from TCGA COAD cohort were divided into the high and low CCC score groups. **(D)** The Kaplan–Meier curve analysis revealed the differential OS between high and low CCC score groups. **(E)** The receiver operating characteristic curve analysis. The decision curve analyses (DCA) of CCC signature in predicting 1-year **(F)**, 2-year **(G)**, and 3-year **(H)** OS for COAD patients.

The univariate Cox regression analysis was conducted to classify the prognostic association between the expression of CCCs and the OS of COAD patients (**Table 1**), and eventually, 27 CCC genes were identified to be significantly correlated with COAD patients’ OS (*p*< 0.05, **Figure 2A**). Subsequently, these survival-related genes were enrolled into the LASSO Cox regression analysis (**Figure 2B**), and a 20-CCC gene signature was constructed (**Supplementary Table S1**). According to the CCC signature scoring system, COAD patients from TCGA cohort were stratified into a high CCC score group and a low CCC score group based on the median CCC score (**Figure 2C**). Patients with higher CCC scores had inferior OS compared to those cases from the low CCC score group (median OS: 57.24 months vs. unreached, *p*< 0.0001, **Figure 2D**). Moreover, the AUC values of CCC signature at 1, 2, and 3 years were 0.74, 0.78, and 0.77, respectively (**Figure 2E**). The decision curve analyses (DCA) further revealed that CCC signature had a better net benefit for OS than TNM staging or clinical staging (**Figures 2F–H**), suggesting that CCC signature was a robust prognosis prediction model.

**Table 1:** Clinical features of 359 patients with COAD from TCGA.

Index		Number
Total		359
Age	Median (range)	66 (31, 90)
Gender	Male	194
	female	165
Histological types	Colon	273
	Rectum	86
Anatomic sites	Left-side colon	123
	Right-side colon	150
T stage	T1	10
	T2	55
	T3	247
	T4	47
N stage	N0	197
	N1	96
	N2	65
M stage	M0	248
	M1	49
Clinical stage	I	55
	II	130
	III	111
	IV	52

COAD, colorectal adenocarcinoma.

The clinical association analyses revealed that there was no statistically significant difference in age at diagnosis or the male or female patient number, which was nearly equivalent between high and low CCC score groups (*p* > 0.05, **Supplementary Figures S1A, B**). Except for T stage (*p* > 0.05, **Supplementary Figure S1C**), patients in the high CCC score group had significantly aggressive clinical phenotypes, such as lymph node invasion and distant metastasis (*p*< 0.05, **Supplementary Figures 1D, E**): 54.74% of COAD patients in high CCC score group had lymph node invasions (N2: 24.02% vs. 12.29%, N1: 30.73% vs. 22.91%, N0: 45.25% vs. 64.80%, *p<* 0.05, **Supplementary Figure S1D**) and 22.6% COAD patients in high CCC score group had metastasis (M1: 22.60% vs. 10.60%, M0: 77.40% vs. 89.40%, *p*< 0.05, **Supplementary Figure S1E**). Furthermore, it was identified that 58.04% COAD patients in high CCC score group had advanced clinical stages, which was significantly higher than those from low CCC score group (stage IV: 20.11% vs. 9.77%, stage III: 37.93% vs. 25.86%, stage II: 30.46% vs. 44.25%, stage I: 11.49% vs. 20.11%, *p*< 0.05, **Supplementary Figure S1F**). Additionally, a comparison of CCC score demonstrated that a higher CCC score was correlated with advanced stages (**Supplementary Figure S2**).

### CCC signature-related genes correlated with clinical features, immune characteristics, and their IHC staining

**Figure 3:**
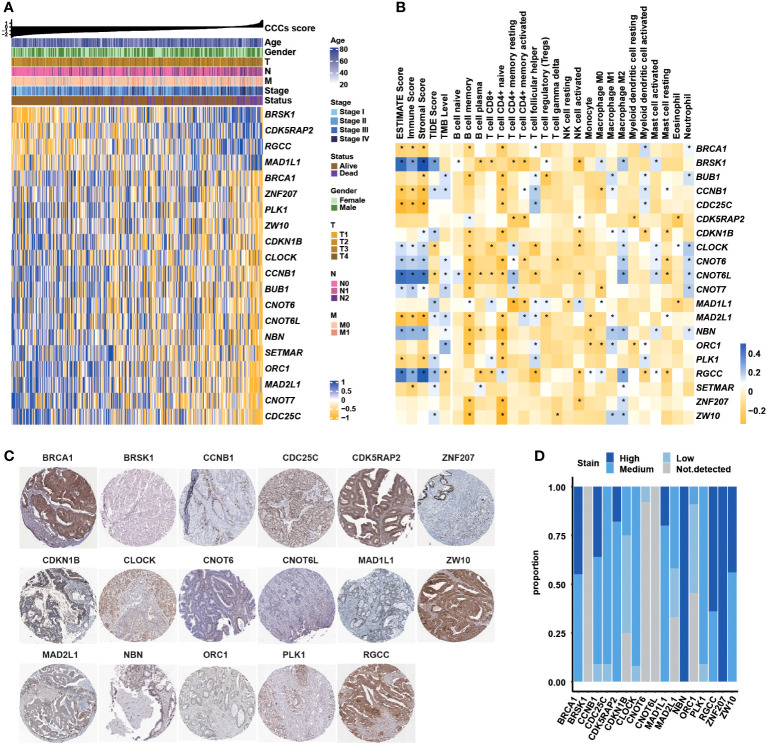
Correlation between the cell cycle checkpoints (CCC) signature score (related genes) with clinical features, immune characteristics, and immunohistochemistry (IHC) staining analysis. **(A)** The heatmap illustrated the correlation between CCC score (related gene expression) and clinical features. **(B)** The correlation analysis between signature-related CCC gene expression and immune-related characteristics, including ESTIMATE score (consisting of the immune score and stromal score), TIDE score, tumor mutational burden (TMB) level, and the infiltration level of 22 immune cells. **(C)** The IHC analysis of signature-related CCC genes. **(D)** The statistical analysis of IHC-related results for signature-related CCC genes.

Obviously, it was observed that *BRSK1*, *CDK5RAP2*, *RGCC*, and *MAD1L1* expression was negatively correlated with TNM stages and clinical stages; in reverse, *BRCA1*, *ZNF207*, *PLK1*, *ZW10*, *CDKN1B*, *CLOCK*, *CCNB1*, *BUB1*, *CNOT6*, *CNOT6L*, *NBN*, *SETMAR*, *ORC1*, *MAD2L1*, *CNOT7*, and *CDC25C* positively associated with advanced stages in COAD (**Figure 3A**). Of note, the expression of a majority of CCC signature-related genes was closely correlated with stromal and immune microenvironment characteristics; meanwhile, the expression of some certain genes was significantly correlated with immune profiles, including TMB, TIDE score, and 22 infiltrating immune cell types (**Figure 3B**). The representative IHC staining of BRCA1, BRSK1, CCNB1, CDC25C, CDK5RAP2, CDKN1B, CLOCK, CNOT6, CNOT6L, MAD1L1, ZW10, MAD2L1, NBN, ORC1, PLK1, RGCC, ZNF207, and ZW10 was shown in **Figure 3C**, while BUB1, CNOT7, and SETMAR protein expression in COAD was not analyzed via the investigation of the HPA database. By further statistical analysis, it was identified that the IHC staining of NBN and ZNF207 protein expression was particularly strong (**Figure 3D**). Except for BRSK1, CNOT6, and CNOT6L, the IHC staining of most of the CCC signature-related genes was moderate.

### Functional verification of signature-related genes in COAD-derived cell lines

**Figure 4:**
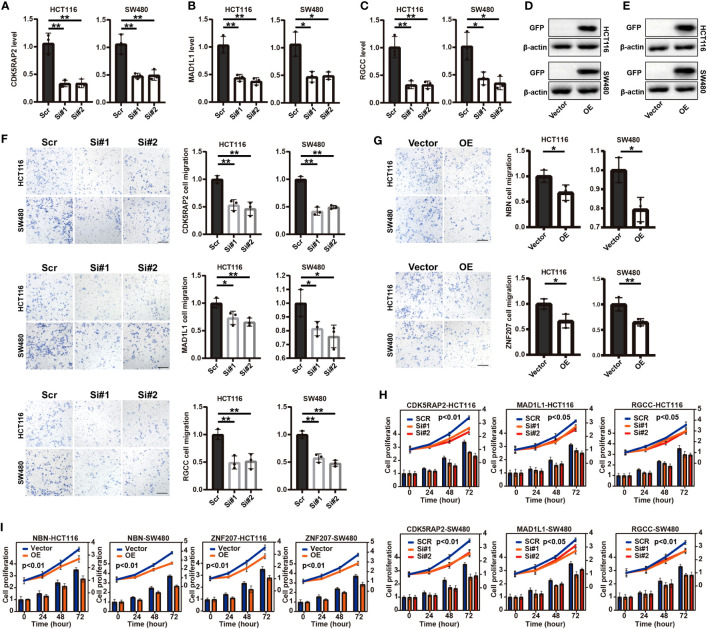
Transwell assay analysis and cell proliferation analysis of five screened (functional role was unknown in COAD) cell cycle checkpoints (CCC) signature-related genes in the COAD-derived cell lines. The qRT-PCR analysis for the verification of the RNA interference by siRNA targeting *CDK5RAP2* **(A)**, *MAD1L1* **(B)**, and *RGCC* **(C)** in the HCT116 and SW480 cell lines. The construction of *NBN*- **(D)** and *ZNF207*-overexpressed **(E)** pCMV plasmid systems in the HCT116 and SW480 cell lines. The Transwell assay analysis demonstrated the effects of the suppressed expression of *CDK5RAP2*, *MAD1L1*, and *RGCC* **(F)** and *NBN* and *ZNF207* overexpression **(G)** on the cell migration of the HCT116 and SW480 cell lines. The effects of the suppressed expression of *CDK5RAP2*, *MAD1L1*, and *RGCC* **(H)** and *NBN* and *ZNF207* overexpression **(I)** on the cell proliferation of HCT116 and SW480 cell lines. *, p<0.05; **, p<0.01.

In the foundation of the literature review, five genes involved in the CCC signature were selected, including the identified risk factors of *CDK5RAP2*, *MAD1L1*, and *RGCC* and the protective factors of *NBN* and *ZNF207*, whose functions were scarcely known in the COAD-derived cell lines. Initially, qRT-PCR analysis confirmed the RNA interference by siRNA targeting *CDK5RAP2*, *MAD1L1*, and *RGCC* (*p*< 0.05, **Figures 4A–C**) in the COAD-derived cancer cell lines of HCT116 and SW480. Meanwhile, Western blotting analysis validated the success of the construction of *NBN*- and *ZNF207*-overexpressed pCMV plasmid systems in the HCT116 and SW480 cell lines (**Figures 4D, E**). Impressively, Transwell assay analysis demonstrated that the downregulation of *CDK5RAP2*, *MAD1L1*, and *RGCC* reduced the cell migration rates of both HCT116 and SW480 cell lines (*p*< 0.05, **Figure 4F**); moreover, *NBN*- and *ZNF207*-overexpressed HCT116 and SW480 cell lines also exhibited the decline of cell migration rates (*p*< 0.05, **Figure 4G**). In addition, it was further observed that the downregulation of *CDK5RAP2*, *MAD1L1*, and *RGCC* decreased cell proliferation levels of both HCT116 and SW480 cell lines (*p*< 0.05, **Figure 4H**). Furthermore, cell proliferation levels were inhibited in the *NBN*- and *ZNF207*-overexpressed HCT116 and SW480 cell lines (*p*< 0.01, **Figure 4I**).

### CCC signature in different clinical stages and histologic subtypes

**Figure 5:**
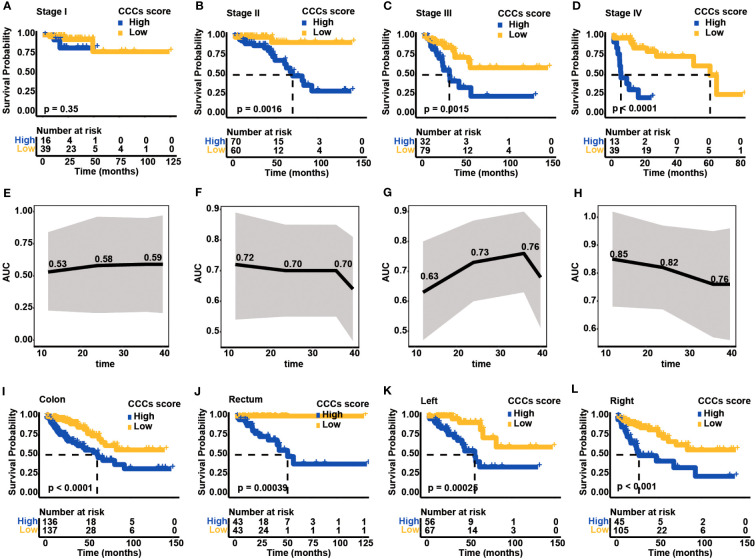
Clinical application of cell cycle checkpoint (CCC) signature. Based on the optimal cutoff value of the CCC score, the Kaplan–Meier curve analysis of the high and low CCC score groups among colorectal carcinoma (COAD) patients in clinical stages I **(A)**, II **(B)**, III **(C)**, and IV **(D)**. The receiver operating characteristic curve analysis of CCC signature in predicting prognosis for COAD patients in clinical stages I **(E)**, II **(F)**, III **(G)**, and IV **(H)**. The application of the CCC signature among patients with colon cancer **(I)** or rectum cancer **(J)**. Regarding colon cancer patients, the Kaplan-Meier curve analysis between the high and low CCC score groups among patients with left-side **(K)** or right-side **(L)** colon cancer.

The prognostic value of the CCC signature in different UICC/AJCC clinical stages was further investigated. Except for COAD patients in clinical stage I (median OS in the high CCC score group vs. low CCC score group: unreached vs. unreached, *p* = 0.35, **Figure 5A**), in clinical stages II, III, and IV patients in high CCC score group had worse OS (median OS in stage II: 70.16 months vs. unreached, *p* = 0.0016; in stage III: 31.96 months vs. unreached, *p* = 0.0015; in stage IV: 5.88 months vs. 61.84 months, *p*< 0.0001, **Figures 5B–D**). In parallel, the above results suggested that the prognostic value of the CCC signature was more robust for patients with advanced stages, and it was further validated by the ROC curve analysis (**Figures 5E–H**). It was revealed that CCC signature had good prognosis prediction for COAD patients in clinical stage II (AUC at 1, 2, and 3 years were 0.72, 0.70, and 0.70, respectively, **Figure 5F**), as well as for patients in clinical stage III (AUC at 1, 2, and 3 years were 0.63, 0.73, and 0.76, respectively, **Figure 5G**). Of note, CCC signature had the best prognosis prediction for COAD patients in clinical stage IV, with the AUC values for OS at 1, 2, and 3 years were 0.85, 0.82, and 0.76, respectively (**Figure 5H**).

Generally, COAD patients from TCGA cohort can be divided into two main types: colon and rectum. As expected, high CCC score group had worse OS both in the colon (median OS: 60.79 months vs. unreached, *p*< 0.0001, **Figure 5I**) and rectum cancer patients (median OS: 57.24 months vs. unreached, *p*< 0.001, **Figure 5J**). According to the colon classification of anatomic sites, the left-side colon part (descending colon, rectosigmoid junction, sigmoid colon, splenic flexure) and right-side colon part (ascending colon, cecum, hepatic flexure, transverse colon) were included in TCGA cohort. Notably, regardless of patients with left- or right-side colon cancer, higher CCC score still indicated a significantly inferior OS (median OS for left-side colon cancer: 56.25 months vs. unreached, *p*< 0.001, **Figure 5K**; for right-side colon cancer: 26.47 months vs. unreached, *p*< 0.001, **Figure 5L**). The above results showed the good performance of CCC signature predicting prognosis in different histological or anatomical subtypes.

### Validation of CCC signature, independent from clinical features

**Figure 6:**
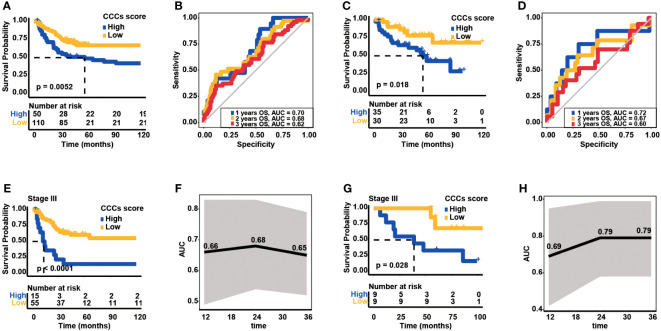
Validation of cell cycle checkpoint (CCC) signature in two additional independent datasets from the Gene Expression Omnibus (GEO) database. **(A)** The Kaplan–Meier curve analysis between the high and low CCC score groups in the GSE24551 dataset. **(B)** The receiver operating characteristic curve analysis of the CCC signature in predicting prognosis for COAD patients in the GSE24551 dataset. **(C)** The Kaplan–Meier curve analysis between the high and low CCC score groups in the GSE29623 dataset. **(D)** The receiver operating characteristic curve analysis of CCC signature in predicting prognosis for COAD patients in the GSE29623 dataset. **(E)** The application of the CCC signature for clinical stage III COAD patients from the GSE24551 dataset. **(F)** The receiver operating characteristic curve analysis of the CCC signature for clinical stage III COAD patients from the GSE24551 dataset. **(G)** The application of the CCC signature for clinical stage III COAD patients from the GSE29623 dataset. **(H)** The receiver operating characteristic curve analysis of the CCC signature for clinical stage III COAD patients from the GSE29623 dataset.

CCC signature was further assessed in two independent cohorts from the GEO database to validate its robustness. In the GSE24551 dataset, the KM curve showed that patients with higher CCC scores had a worse OS (median OS: 57.00 months vs. unreached, *p*< 0.01, **Figure 6A**), and the AUC values of CCC signature for OS at 1, 2, and 3 years were 0.70, 0.68, and 0.62, respectively (**Figure 6B**). Likewise, in the GSE29623 dataset, there was a lower OS probability of patients from the high CCC score group (median OS: 54.90 months vs. unreached, *p*< 0.05, **Figure 6C**), and the AUC values of CCC signature for OS at 1, 2, and 3 years were 0.72, 0.67, and 0.60, respectively (**Figure 6D**). In addition, the prognostic capability of CCC signature for advanced COAD patients was also verified in these two validation cohorts. Regarding COAD patients in clinical stage III from the GSE24551 dataset, it could be obviously seen that patients in the high CCC score group also had a shorter OS (median OS: 13.02 months vs. unreached, *p*< 0.0001, **Figure 6E**), and the AUC values for OS at 1, 2, and 3 years were 0.66, 0.68, and 0.65, respectively (**Figure 6F**). Similarly, for patients in clinical stage III from GSE29623 dataset, higher CCC score indicated poorer OS (median OS: 38.72 months vs. unreached, *p*< 0.05, **Figure 6G**), and the AUC values for OS at 1, 2, and 3 years were 0.69, 0.79, and 0.79, respectively (**Figure 6H**). More importantly, the univariate and multivariate Cox proportional hazards regression analyses (**Table 2**) further identified that the CCC signature was independent of clinical features (*p*< 0.001) in predicting prognosis for COAD patients.

**Table 2:** Univariate and multivariate Cox proportional hazard regression analyses based on CCC score and clinical features in TCGA COAD cohort.

Variables	Univariate			Multivariate		
	HR	95% CI	*p*-value	HR	95% CI	*p*-value
CCC score						
High/Low	3.28	1.99–5.40	< 0.01^**^	3.30	1.83–5.95	< 0.01^**^
Age						
> 66/< 66	2.36	1.45–3.83	<0.01^**^	4.01	2.23–7.22	<0.01^**^
Sex						
Male/Female	1.16	0.75–1.81	0.51	0.60	0.36–1.00	0.05
T stage						
T3 and T4/T1 and T2	2.21	1.02–4.82	< 0.05^*^	1.50	0.52–4.31	0.45
N stage						
N1 and N2/N0	2.45	1.56–3.87	< 0.01^**^	0.57	0.17–1.89	0.36
M stage						
M1/M0	3.38	1.98–5.78	< 0.01^**^	3.11	1.63–5.94	< 0.01^**^
Clinical stage						
III and IV/I and II	2.71	1.68–4.37	< 0.01^**^	3.51	0.96–12.9	0.06

CCCs, cell cycle checkpoints; COAD, colorectal adenocarcinoma; HR, hazard ratio; CI, confidence interval. *, p<0.05; **, p<0.01.

### Prognostic value of CCC signature in distinct molecular subtypes

Subsequently, the prognostic value of the CCC signature in different mutant subgroups was explored. In *APC*-mutant, *TP53*-mutant, *KRAS/NARS/BRAF*-mutant subgroups and along with corresponding wild subgroups, respectively, it was also found that higher CCC score was always correlated with the poorer clinical outcomes (median OS in *APC*-wild subgroup: 67.29 months vs. 70.16 months, *p*< 0.05; in *APC*-mutant subgroup: 23.51 months vs. unreached, *p*< 0.0001; in *TP53*-wild subgroup: 81.37 months vs. unreached, *p*< 0.001; in *TP53*-mutant subgroup: 19.66 months vs. 92.74 months, *p*< 0.0001; in *KRAS/NARS/BRAF*-wild subgroup: 44.32 months vs. unreached, *p*< 0.001; in *KRAS/NARS/BRAF*-mutant subgroup: 16.80 months vs. unreached, *p*< 0.0001, **Supplementary Figures S3A–F**). Similarly, no matter whether patients were in MSI-H or MSI-L and MSS subgroups, the high CCC score group had lower OS probability (median OS in MSI-H subgroup: 70.16 months vs. unreached, *p* = 0.071; in MSI-L and MSS subgroup: 22.42 months vs. unreached, *p*< 0.0001, **Supplementary Figures S3G, H**). Regarding the CMS molecular subtypes (CMS1, *N* = 29; CMS2, *N* = 68; CMS3, *N* = 58; CMS4, *N* = 114), it was initially found that patients in the CMS3 subgroup had the best OS (median OS of CMS4 vs. CMS3 vs. CMS2 vs. CMS1: 65.85 months vs. unreached vs. 49.41 months, *p*< 0.05, **Supplementary Figure S4A**). Accordingly, a higher proportion of CMS3 subtype patients could be assigned into the low CCC score group (**Supplementary Figure S4B**), and impressively, CMS3 subgroup had the significantly lowest CCC score than those of other subgroups (*p*< 0.0001, **Supplementary Figure S4C**). Even on the CMS stratification, CCC signature still performed well in the prognosis prediction, and the KM curve analysis demonstrated that the high CCC score group always had the worse OS in each CMS subgroup (median OS in CMS1: 38.07 months vs. unreached, *p*< 0.01; in CMS2: 41.36 months vs. unreached, *p*< 0.05; in CMS3: unreached vs. unreached, *p* = 0.097; in CMS4: 22.42 months vs. 65.85 months, *p*< 0.001, **Supplementary Figures S4D–G**).

### Molecular characteristics of different CCC score subgroups

**Figure 7:**
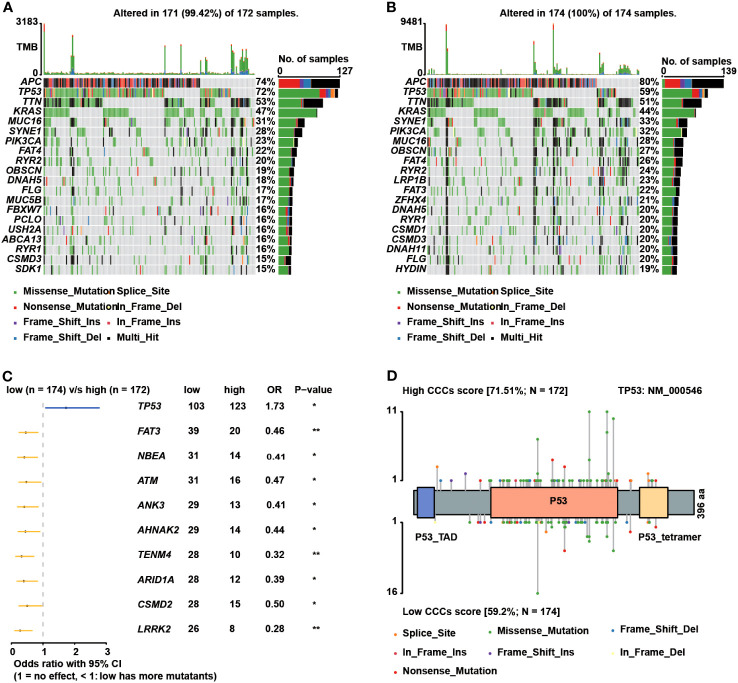
The genomic differences between the high and low cell cycle checkpoints (CCC) score groups. The oncoprint plots exhibited the top 20 altered genes in the high **(A)** and low **(B)** CCC score groups, respectively. **(C)** The forest plot deciphered the prevalent genes in the high and low CCC score groups. **(D)** The lollipop plot showed the prevalence of altered sites in the high and low CCC score groups.

**Figure 8:**
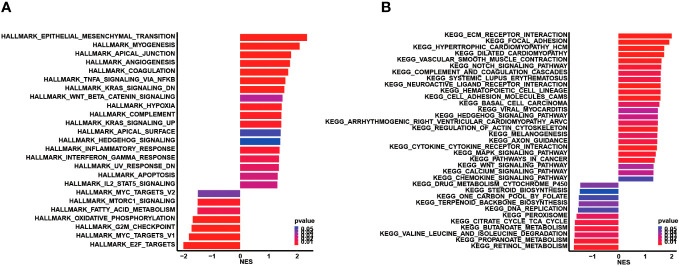
Functional enrichment analysis based on the differentially expressed genes between the high and low CCC score groups. **(A)** The HALLMARK pathway enrichment analysis. **(B)** The KEGG pathway enrichment analysis.

Subsequently, genomic alteration profiling was conducted to uncover the genomic difference between high and low CCC score groups in TCGA cohort. As shown, nearly all COAD patients had at least one genomic mutation event, consistent with previous studies, no matter whether in a high CCC score group or a low CCC score group (total mutation frequency in high CCC score group vs. in low CCC score group: 99.42% vs. 100%). The genomic mutation profile of the top 20 altered genes demonstrated that *APC*, *TP53*, *TTN*, and *KRAS* were the most prevalently altered genes in both groups (**Figures 7A, B**). By further statistical analysis, it was identified that only *TP53* was prevalently altered in the high CCC score group (*p*< 0.05, **Figure 7C**); whereas, *FAT3*, *NBEA*, *ATM*, *ANK3*, *AHNAK2*, *TENM4*, *ARID1A*, *CSMD2*, and *LRRK2* were more frequently altered in the low CCC score group (*p*< 0.05, **Figure 7C**). Furthermore, the lollipop plot further displayed that the altered site p.R273C was more prevalent in the high CCC score group (*p*< 0.05, **Figure 7D**). Additionally, HALLMARK and KEGG pathway enrichment analyses based on the DEGs between high- and low-risk groups (**Figure 8**) revealed that extracellular matrix (ECM)-related signaling, angiogenesis, cell cycle-related signaling pathways, immune (chemokine)-related signaling pathways, valine leucine and isoleucine degradation, citrate cycle_TCA cycle, butanoate and propanoate metabolisms were significantly enriched.

### Immune features and anticancer immunity underlying CCC signature

**Figure 9:**
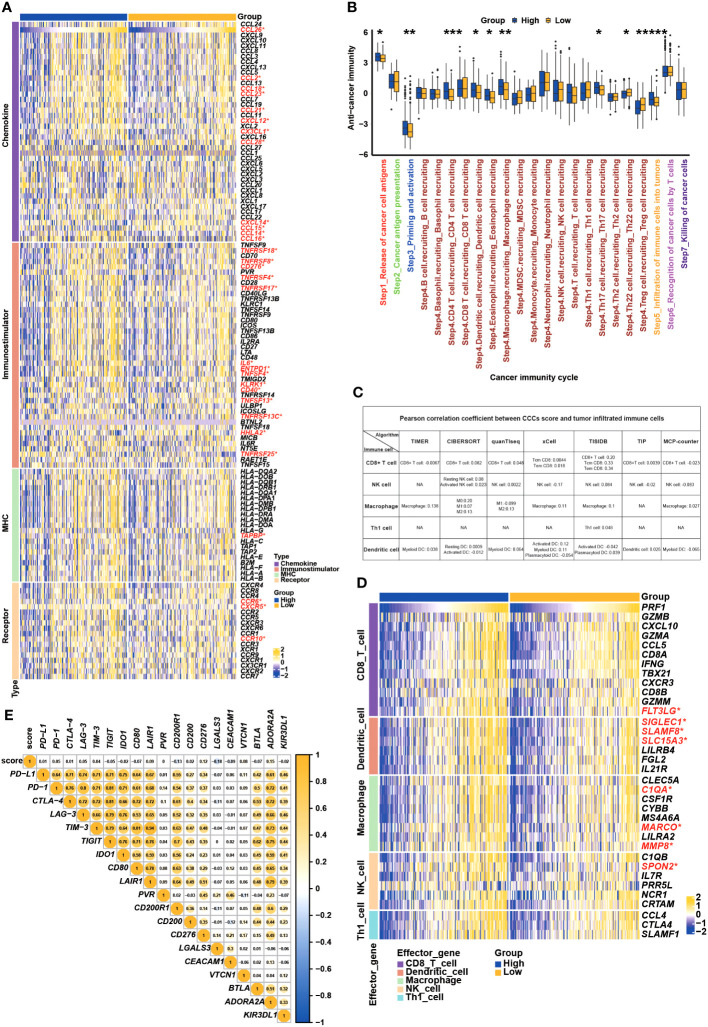
Underlying cell cycle checkpoints (CCC) signature, immune features, and anti-cancer immunity analysis. **(A)** The differentially expressed genes analysis of chemokines, immunostimulators, MHC molecules, and receptors between the high and low CCC score groups. **(B)** The comparison analysis of anticancer immunity cycle activities between the high and low CCC score groups. **(C)** Multiple algorithms were employed to investigate the relationship between the CCC score and the infiltration level of immune-related cells. **(D)** Correlation analysis between CCC score and the effector gene expression of CD8+ T cells, dendritic cells, macrophages, NK cells, and Th1 cells. **(E)** Correlate of CCC score with the expression of inhibitory immune checkpoints. *, p<0.05; **, p<0.01; ***, p<0.001; ****, p<0.0001.

Subsequently, the immunological regulation underlying the CCC signature was deeply investigated in TCGA COAD cohort. As demonstrated, the expression of a majority of chemokines and paired receptors (*CCL2*, *CCL14*, *CCL16*, *CCL18*, *CCL21*, *CCL23*, *CCL26*, *CCL28*, *CXCL12*, *CX3CL1*, *CCR6*, *CCR10*, and *CXCR5*) was significantly higher in the high CCC score group when compared to the low CCC score group (*p*< 0.05, **Figure 9A**). Most immunostimulators (*TNFSF4*, *TNFSF13*, *TNFRSF4*, *TNFRSF8*, *TNFRSF13*, *TNFRSF17*, *TNFRSF18*, *TNFRSF25*, *TNFRSF13C*, *IL6*, *ENTPD1*, *KLRK1*, *CD40*, and *CD276*) were also markedly upregulated in the high CCC score group. Whereas, it was identified that only *TAPBP* (MHC-associated gene) expression was significantly elevated in the high CCC score group. In short, COAD patients in the high CCC score group might have an immune-promoting microenvironment compared to those in the low CCC score group. Correspondingly, the high CCC score group had a higher level of most of the anticancer immunity cycle activities, including step 1 of cancer cell antigen release, step 3 of priming and activation, step 4 (trafficking to tumors) of CD4 T cell, dendritic cell, eosinophil, macrophage, and Th17 cell recruiting, and step 5 of infiltration of immune cells into tumors (*p*< 0.05, **Figure 9B**). By different algorithms, including TIMER, CIBERSORT, QUANTISEQ, XCELL, TISIDB, TIP, and MCPCOUNTER (**Figure 9C**), it was found that there was nearly no correlation between the CCC score and the infiltrating levels of macrophages, dendritic cells, NK cells, and CD8+ T cells. However, some effector genes (such as CD8 T cell: *FLT3LG*; dendritic cell: *SIGLEC1*, *SLAMF8*, and *SLC15A3*; macrophage: *C1QA*, *MARCO*, and *MMP8*; natural killing cell: *SPON2*) expression was significantly upregulated in the high CCC score group (**Figure 9D**). In addition, it was further identified that the CCC score was not closely associated with *PD-L1*, *PD-1*, *CTLA-4*, *LAG-3*, *TIM-3*, *TIGHT*, *IDO1*, and other inhibitory immune checkpoints at the transcriptomic level (**Figure 9E**).

### CCC signature in the prediction of therapeutic response

**Figure 10:**
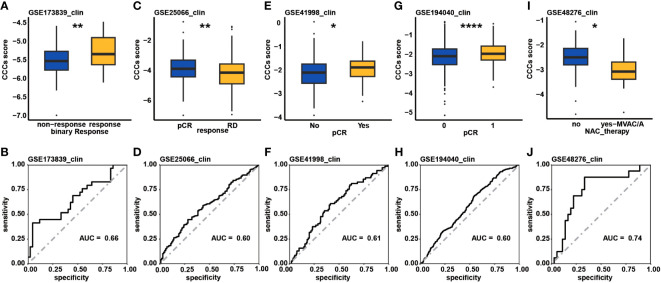
The therapeutic response prediction underlying cell cycle checkpoints (CCC) signature. Comparison analysis of CCC score between the response and non-response patient groups in the GSE173839 **(A)**, GSE25066 **(C)**, GSE41998 **(E)**, and GSE194040 **(G)** datasets. The receiver operating characteristic curve analysis of CCC signature in predicting therapeutic response in the GSE173839 **(B)**, GSE25066 **(D)**, GSE41998 **(F)**, and GSE194040 **(H)** datasets. **(I)** In the GSE48276 dataset, patients had a significantly decreased CCC score after receiving the frontline chemotherapy. **(J)** The receiver operating characteristic curve analysis of CCC signature in predicting therapeutic response in the GSE48276. *, p<0.05; **, p<0.01; ****, p<0.0001.

Eventually, the predictive role of the CCC signature in the therapeutic response was investigated. In the GSE153412 dataset, including HCT116 & HT29 cell lines treated by the chemotherapy or radiochemotherapy, it was identified that the low CCC score group seemed to be likely more sensitive to 5-fluorouracil (5-FU), uracil, or radiochemotherapy of 5-FU and irradiation (*p*< 0.01, **Supplementary Figure S5A**). In the GSE196263 dataset, five colon cancer patients belonging to the CMS4 subtype had a significantly reduced CCC score after imatinib treatment (*p*< 0.01, **Supplementary Figure S5B**). In the GSE173839 dataset of HER2-negative stage II/III breast cancer patients on the durvalumab with olaparib and paclitaxel arm, it seemed that higher CCC score was correlated with pCR (*p*< 0.01, **Figure 10A**). Impressively, the AUC value of the CCC score for response prediction was 0.66 (**Figure 10B**). In another breast cancer dataset of GSE25066, a higher CCC score was also correlated with the pCR of patients receiving taxane-anthracycline chemotherapy (*p*< 0.01, **Figure 10C**), and the AUC value for response prediction was 0.60 (**Figure 10D**). Moreover, it was further found that the CCC score was positively correlated with the pCR (*p*< 0.05, **Figure 10E**) of neoadjuvant cyclophosphamide/doxorubicin with ixabepilone or paclitaxel for early-stage breast cancer patients in the GSE41998 dataset, and the AUC value for response prediction was 0.61 (**Figure 10F**). A recent study of the I-SPY2-990 mRNA/RPPA Data Resource containing gene expression and clinical data for 987 patients from 10 arms of the neoadjuvant I-SPY2 TRIAL for aggressive early-stage breast cancer (GSE194040) found that, interestingly, a higher CCC score was positively correlated with the pCR of neoadjuvant chemotherapy (*p*< 0.0001, **Figure 10G**), and the AUC value for response prediction was 0.60 (**Figure 10H**). Whereas, in the GSE48276 dataset of bladder cancer patients, those cases had a significantly decreased CCC score after receiving the frontline chemotherapy (**Figure 10I**), and the AUC value was 0.74 (**Figure 10J**).

### Evaluation of chemotherapeutic response underlying CCC signature

**Figure 11:**
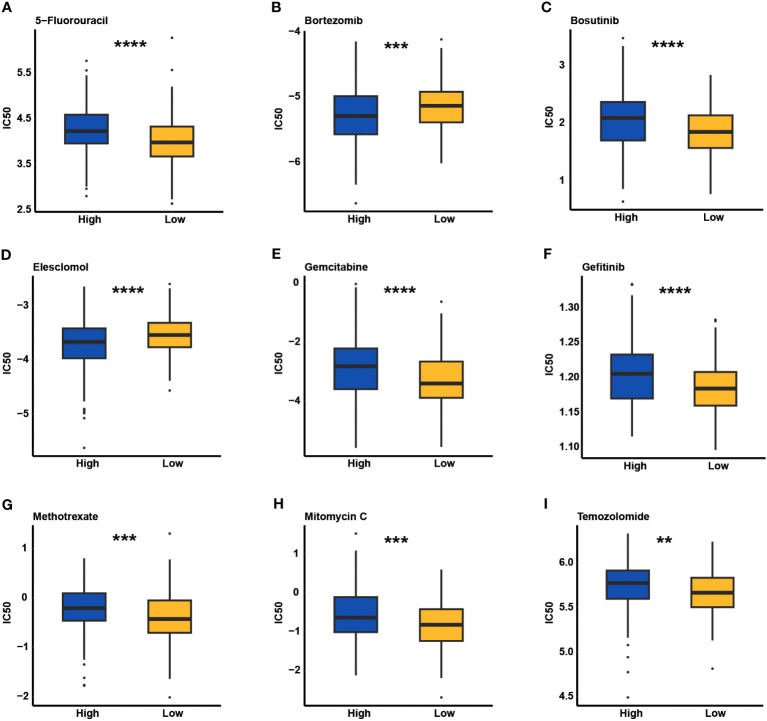
The estimate of chemotherapeutic response underlying cell cycle checkpoints (CCCs) signature in the Genomics of Drug Sensitivity in Cancer (GDSC) database. The IC50 value evaluation of 5-fluorouracil **(A)**, bortezomib **(B)**, bosutinib **(C)**, elesclomol **(D)**, gemcitabine **(E)**, gefitinib **(F)**, methotrexate **(G)**, mitomycin C **(H)**, and temozolomide **(I)** between the high and low CCC score groups. **, p<0.05; ***, p<0.001; ****, p<0.0001.

Through the analysis of the GDSC database, the predictive value of the CCC signature for chemotherapy response was estimated. Comparison analysis of the IC50 value revealed that patients with a lower CCC score were likely to be more sensitive to 5-fluorouracil, bosutinib, gemcitabine, gefitinib, methotrexate, mitomycin C, and temozolomide (*p*< 0.01, **Figure 11**). Whereas, patients with higher CCC score seemed to have the higher level of sensitivity to bortezomib and elesclomol (*p*< 0.001, **Figure 11**).

## Discussions

Substantial evidence shows that CRC is a kind of heterogeneous cancer, for which the prognosis and the efficacy of treatment strategies vary. Especially for most of the CRC patients with advanced/metastatic disease, chemotherapy, radiation therapy, and targeted therapy become less effective and the 5-year survival rate is notably lower (49). Molecular profiling, such as genomic profiling, transcriptomic profiling, and proteomic profiling, is now a major objective to classify heterogeneous CRC patients and guide personalized treatment (50). In our study, the mRNA expression profiling of a total of 209 CCCs was investigated, and finally, a novel CCC signature based on 20 CCC-related genes was constructed along with a scoring system that performed robustly in the prognosis prediction for COAD patients, especially for advanced patients. Also, the CCC signature also had good risk stratification in different subgroups with distinct clinical features or molecular characteristics. The differentiation analysis of biological functions and TMEs between high and low CCC score groups further revealed the underlying molecular characterization and immunological regulation in COAD, which could guide clinical decision-making. Moreover, the CCC signature was also of considerable guiding significance in predicting therapeutic responses for COAD patients. Collectively, the CCC signature had the potential to improve the COAD management in clinical practices.

Among these identified CCC genes, the aberrant regulation of *BRCA1* (51), *BRSK1* (52), *BUB1* (53), *CCNB1* (54), *CDC25C* (55), *CDKN1B* (56), *CLOCK* (57), *CNOT6* and *CNOT6L* (58), *CNOT7* (59), *MAD2L1* (60), *ORC1* (61), *PLK1* (62), *SETMAR* (63), and *ZW10* (64) has been found to exert influences on CRC cell lines or the prognosis of colon or rectum cancer patients. Of note, it is the first time it has been identified that *CDK5RAP2*, *MAD1L1*, *NBN*, *RGCC*, and *ZNF207* expressions are markedly correlated with the prognosis of CRC patients. In the present study, the functional roles of risk factors *CDK5RAP2*, *MAD1L1*, and *RGCC* and protective factors *NBN* and *ZNF207* were deeply investigated through cell proliferation and Transwell assay analysis. From another perspective, it could be inferred that inhibition of *CDK5RAP2*, *MAD1L1*, and *RGCC* expression could suppress the proliferation, migration, and invasion of COAD cells, which might be an effective treatment strategy for COAD patients. Furthermore, according to the CCC signature scoring system, the integrative role of cell cycle checkpoint mechanisms as the risk factor in COAD was quantified, and the constructed CCC signature had a good ability of stratifying COAD patients into different risk groups in various TNM/clinical stages, histological subtypes, or molecular subtypes, allowing the molecular characterization of COAD progression.

Considerable studies have revealed that continuous cancer cells are generally driven by mutations, not only compromising cell cycle exit but also preventing apoptosis, rather than promoting boundless cell cycle progression. During processing, the checkpoint mechanisms strictly control cell cycle entry and exit and regulate cell cycle progression (22). As for reversible or irreversible DNA damage, the checkpoint mechanisms could function to initiate quiescence or induce apoptosis or senescence, respectively (65). Nevertheless, in cancer cells, compromised checkpoints could tolerate chromosome instability and aneuploidy; however, too much is harmful that could suppress cancer cell growth and promote cell death instead (66, 67), and high level of genomic alterations has been identified to be correlated with the improved prognosis of cancer patients (68). Generally, DNA damage checkpoints function in response to irreversible DNA damage mostly by *TP53*-dependent pathways, but it should be highlighted that p53 inactivation prominently led to the suppression of cell cycle arrest and apoptosis (65). Therefore, the aberrant regulation of cell cycle checkpoints (DNA damage checkpoints) would promote uncontrolled cell cycle progression. Moreover, this study simultaneously used two cell lines, SW480 (a p53 mutated cell line) and HCT116 (a p53 wildtype cell line), to try to prove that the aberrant regulation of CCC signature-related gene(s) expression was relatively independently correlated with the proliferation, progression, and invasion of colorectal cancer cells. Unsurprisingly, our finding also revealed the higher prevalence of the *TP53* mutation (72% vs. 59%) in the high CCC score group. The characterization of genomic alterations in CRC by TCGA have already revealed that *APC*, *TP53*, *SMAD4*, *PIK3CA*, and *KRAS* mutations were most prevalent (69), in which *APC*, *TP53*, and *KRAS/NARS/BRAF* mutations have been found to be highly predictive for outcomes in clinical practice (41, 70). Therefore, the prognostic role of the CCC signature in different mutant subgroups was investigated, and remarkably, a lower CCC score always indicated a superior OS.

Functional enrichment of the KEGG pathway underlying the CCC signature revealed that ECM-related signaling pathways, angiogenesis, cell cycle-related signaling pathways, immune (chemokine)-related signaling pathways, valine leucine and isoleucine degradation, citrate cycle_TCA cycle, butanoate, and propanoate metabolisms were enriched. Previous study has found that the downregulation of butanoate and propanoate metabolism and valine, leucine, and isoleucine degradation happened in the metastatic tissues from all sites in metastatic colon patients, and metastatic tissues from the liver and omentum had the decreased regulation of oxidative phosphorylation (71). The increased intake of branched-chain amino acids, such as valine, leucine, and isoleucine, was positively correlated with the mortality rate of CRC patients (72). Moreover, in the mouse model, it was found that mirabilite exerted a therapeutic effect on CRC, and the significantly enriched propanoate metabolism was one of the therapeutic targets (73). Furthermore, the high-throughput metabolomic analysis disclosed that an active polyphenolic honokiol (HNK) had anticancer activities mainly by regulating the citrate cycle_TCA cycle, tryptophan metabolism, and pentose phosphate pathway in the mouse model (74). Except as a diagnostic biomarker in CRC (75), the role of butanoate and related metabolism in CRC progression is unknown until now. Overall, CCC inhibitors, together with the controlled level of valine, leucine, and isoleucine, and in combination with the targeted therapeutics for propanoate and butanoate metabolisms as well as citrate cycle_TCA cycle, might help improve the prognosis of COAD patients with specific subtypes. However, the underlying mechanisms of cell cycle checkpoints regulating metabolisms are scarcely known and need to be further verified.

Frequently, immunomodulators, cancer immunity, tumor immune cell infiltration, and inhibitory immune checkpoints were used to evaluate the immunological status of TME (76). A majority of chemokines and paired receptors could stimulate the recruitment of CD8+ T cells in human cancers (45, 77–79). As shown, these downregulated chemokine/receptors among patients with lower CCC scores would reduce the activity of anticancer immunity. Impressively, patients with higher CCC scores seemed to have an immune-promoting microenvironment, owing to the significantly upregulated expression of a majority of chemokines, receptors, and immunostimulators. Comprehensively, the complex functions and interactions of these immunomodulators could be integrated and reflected by seven major steps, directly representing the anticancer immunity of tumor cells (46). As further confirmed, patients with higher CCC scores had a higher level of most of the anticancer immunity cycle activities, including step 1 of cancer cell antigen release, step 3 of priming and activation, step 4 (trafficking to tumors) of CD4 T cell, dendritic cell, eosinophil, macrophage, and Th17 cell recruiting, and step 5 of infiltration of immune cells into tumors. Indeed, an immune-promoting microenvironment should have improved the prognosis of COAD patients; however, the anticancer immunity cycle of step 6 of recognition of cancer cells by T cells and step 7 of the killing of cancer cells between high and low CCC score groups were nearly equivalent. Thus, it could be inferred that this antitumor effect in the high CCC score group might be restrained by tumor immune escape, owing to the higher level of PD-1 and/or PD-L1. In brief, the CCC score could be of guiding significance to clarifying COAD patients with an immunosuppressive or immune-promoting microenvironment, which would help predict the response of immunotherapy in COAD.

Ultimately, this study further investigated the role of CCC signature in the prediction of therapeutic response. Regarding the analysis of the GSE153412 dataset of HCT116 and HT29 cell lines treated with chemotherapy or radiochemotherapy, it was suggested that the CCC signature might distinguish a patient subgroup, with the low CCC score being likely more sensitive to chemotherapy of 5-FU/uracil or radiochemotherapy. Notably, it was identified in the present study that COAD patients belonging to the CMS4 subtype might have improved OS after imatinib treatment because the CCC score was markedly reduced. Imatinib, as a kind of inhibitor-targeting tyrosine kinase, already exhibited its effects of inhibiting the proliferation of stromal fibroblasts and preventing colorectal metastases (80). In spite of that, more clinical trials were needed to validate the efficacy of imatinib treatment for CMS4 subtype COAD patients, while the CCC signature seemed to be a good biomarker to predict the treatment efficacy of imatinib. Furthermore, the analysis results of GSE173839, GSE25066, GSE41998, and GSE194040 datasets underlying CCC signature suggested that durvalumab with olaparib and paclitaxel, taxane-anthracycline chemotherapy, neoadjuvant cyclophosphamide/doxorubicin with ixabepilone or paclitaxel, and immunotherapeutic strategies might also be suitable for COAD patients with a higher CCC score. Altogether, the present study recommended more potential treatment selections for COAD patients with distinct molecular characteristics. Whereas, more experimental explorations and clinical trials are urgently needed to verify the related analysis results in this research.

## Conclusion

The novel CCC signature had good sensitivity and specificity as a robust prognostic model, regardless of clinical features, histological subtypes, or molecular subgroups, which could help promote the clinical management of COAD. Furthermore, the CCC signature distinguished different groups with distinct molecular features, immune-related characteristics, and therapeutic responses, which would help advance precision medicine for COAD patients.

## Data availability statement

The original contributions presented in the study are included in the article/**Supplementary Material**, further inquiries can be directed to the corresponding authors.

## Author contributions

HW: Conceptualization, Data curation, Formal Analysis, Investigation, Methodology, Resources, Visualization, Writing – original draft. WW: Data curation, Formal Analysis, Investigation, Methodology, Resources, Software, Validation, Visualization, Writing – original draft. ZW: Conceptualization, Formal Analysis, Investigation, Methodology, Resources, Validation, Visualization, Writing – original draft, Writing – review & editing. XL: Conceptualization, Funding acquisition, Investigation, Project administration, Supervision, Validation, Writing – original draft, Writing – review & editing.

## References

[1] Bray F Ferlay J Soerjomataram I Siegel RL Torre LA Jemal A. Global cancer statistics 2018: GLOBOCAN estimates of incidence and mortality worldwide for 36 cancers in 185 countries. CA: Cancer J Clin (2018) 68(6):394–424. doi: 10.3322/caac.21492 30207593

[2] Kanth P Inadomi J. Screening and prevention of colorectal cancer. BMJ (Clinical Res ed.) (2021) 374:n1855. doi: 10.1136/bmj.n1855 34526356

[3] Weitz J Koch M Debus J Höhler T Galle PR Büchler MW. Colorectal cancer. Lancet (London England) (2005) 365(9454):153–65. doi: 10.1016/S0140-6736(05)17706-X 15639298

[4] Weiser M. AJCC 8th edition: colorectal cancer. Ann Surg Oncol (2018) 25(6):1454–5. doi: 10.1245/s10434-018-6462-1 29616422

[5] Ten Hoorn S de Back TR Sommeijer DW Vermeulen L. Clinical value of consensus molecular subtypes in colorectal cancer: A systematic review and meta-analysis. J Natl Cancer Institute (2021) 114:503–16. doi: 10.1093/jnci/djab106 PMC9002278 34077519

[6] Mukherji R Marshall J Seeber A. Genomic alterations and their implications on survival in nonmetastatic colorectal cancer: status quo and future perspectives. Cancers (2020) 12(8):1–22. doi: 10.3390/cancers12082001 PMC7465976 32707813

[7] Hauptman N Skok DJ Spasovska E Boštjančič E Glavač D. Genes CEP55, FOXD3, FOXF2, GNAO1, GRIA4, and KCNA5 as potential diagnostic biomarkers in colorectal cancer. BMC Med Genomics (2019) 12(1):54. doi: 10.1186/s12920-019-0501-z 30987631 PMC6466812

[8] Kagohara L Stein-O'Brien GL Kelley D Flam E Wick HC Danilova LV. Epigenetic regulation of gene expression in cancer: techniques, resources and analysis. Briefings Funct Genomics (2018) 17(1):49–63. doi: 10.1093/bfgp/elx018 PMC5860551 28968850

[9] Baylin S Jones P. Epigenetic determinants of cancer. Cold Spring Harbor Perspect Biol (2016) 8(9):1–35. doi: 10.1101/cshperspect.a019505 PMC5008069 27194046

[10] Chisanga D Keerthikumar S Pathan M Ariyaratne D Kalra H Boukouris S. Colorectal cancer atlas: An integrative resource for genomic and proteomic annotations from colorectal cancer cell lines and tissues. Nucleic Acids Res (2016) 44:D969–74. doi: 10.1093/nar/gkv1097 PMC4702801 26496946

[11] Jiang Y Casey G Lavery IC Zhang Y Talantov D Martin-McGreevy M. Development of a clinically feasible molecular assay to predict recurrence of stage II colon cancer. J Mol diagnostics JMD (2008) 10(4):346–54. doi: 10.2353/jmoldx.2008.080011 PMC2438204 18556775

[12] O'Connell M Lavery I Yothers G Paik S Clark-Langone KM Lopatin M. Relationship between tumor gene expression and recurrence in four independent studies of patients with stage II/III colon cancer treated with surgery alone or surgery plus adjuvant fluorouracil plus leucovorin. J Clin Oncol (2010) 28(25):3937–44. doi: 10.1200/JCO.2010.28.9538 PMC2940392 20679606

[13] Salazar R Roepman P Capella G Moreno V Simon I Dreezen C. Gene expression signature to improve prognosis prediction of stage II and III colorectal cancer. J Clin Oncol (2011) 29(1):17–24. doi: 10.1200/JCO.2010.30.1077 21098318

[14] Kennedy R Bylesjo M Kerr P Davison T Black JM Kay EW. Development and independent validation of a prognostic assay for stage II colon cancer using formalin-fixed paraffin-embedded tissue. J Clin Oncol (2011) 29(35):4620–6. doi: 10.1200/JCO.2011.35.4498 22067406

[15] Zhu J Deane NG Lewis KB Padmanabhan C Washington MK Ciombor KK. Evaluation of frozen tissue-derived prognostic gene expression signatures in FFPE colorectal cancer samples. Sci Rep (2016) 6:33273. doi: 10.1038/srep33273 27623752 PMC5021945

[16] Guinney J Dienstmann R Wang X de Reyniès A Schlicker A Soneson C. The consensus molecular subtypes of colorectal cancer. Nat Med (2015) 21(11):1350–6. doi: 10.1038/nm.3967 PMC4636487 26457759

[17] Dienstmann R Vermeulen L Guinney J Kopetz S Tejpar S Tabernero J. Consensus molecular subtypes and the evolution of precision medicine in colorectal cancer. Nat Rev Cancer (2017) 17(2):79–92. doi: 10.1038/nrc.2016.126 28050011

[18] Dunne P McArt DG Bradley CA O'Reilly PG Barrett HL Cummins R. Challenging the cancer molecular stratification dogma: intratumoral heterogeneity undermines consensus molecular subtypes and potential diagnostic value in colorectal cancer. Clin Cancer Res (2016) 22(16):4095–104. doi: 10.1158/1078-0432.CCR-16-0032 27151745

[19] Dunne P Alderdice M O'Reilly PG Roddy AC McCorry AMB Richman S. Cancer-cell intrinsic gene expression signatures overcome intratumoural heterogeneity bias in colorectal cancer patient classification. Nat Commun (2017) 8:15657. doi: 10.1038/ncomms15657 28561046 PMC5460026

[20] Ahluwalia P Kolhe R Gahlay G. The clinical relevance of gene expression based prognostic signatures in colorectal cancer. Biochim Biophys Acta Rev Cancer (2021) 1875(2):188513. doi: 10.1016/j.bbcan.2021.188513 33493614

[21] Barr A Mansfeld J. FEBS letters special issue: cell cycle control. FEBS Lett (2019) 593(20):2803–4. doi: 10.1002/1873-3468.13638 31657016

[22] Matthews H Bertoli C de Bruin R. Cell cycle control in cancer. Nat Rev Mol Cell Biol (2021) 23:74–88. doi: 10.1038/s41580-021-00404-3 34508254

[23] Fernando M Duijf PHG Proctor M Stevenson AJ Ehmann A Vora S. Dysregulated G2 phase checkpoint recovery pathway reduces DNA repair efficiency and increases chromosomal instability in a wide range of tumours. Oncogenesis (2021) 10(5):41. doi: 10.1038/s41389-021-00329-8 33993200 PMC8124070

[24] Neizer-Ashun F Bhattacharya R. Reality CHEK: Understanding the biology and clinical potential of CHK1. Cancer Lett (2021) 497:202–11. doi: 10.1016/j.canlet.2020.09.016 32991949

[25] Li G Zhang H. Mad2 and p27 expression profiles in colorectal cancer and its clinical significance. World J Gastroenterol (2004) 10(21):3218–20. doi: 10.3748/wjg.v10.i21.3218 PMC4611278 15457580

[26] Li G Li H Zhang H. Mad2 and p53 expression profiles in colorectal cancer and its clinical significance. World J Gastroenterol (2003) 9(9):1972–5. doi: 10.3748/wjg.v9.i9.1972 PMC4656655 12970887

[27] Hernando E Nahlé Z Juan G Diaz-Rodriguez E Alaminos M Hemann M. Rb inactivation promotes genomic instability by uncoupling cell cycle progression from mitotic control. Nature (2004) 430(7001):797–802. doi: 10.1038/nature02820 15306814

[28] Ryan S Britigan EMC Zasadil LM Witte K Audhya A Roopra A. Up-regulation of the mitotic checkpoint component Mad1 causes chromosomal instability and resistance to microtubule poisons. Proc Natl Acad Sci United States America (2012) 109(33):E2205–14. doi: 10.1073/pnas.1201911109 PMC3421180 22778409

[29] Danielsen SA Cekaite L Ågesen TH Sveen A Nesbakken A Thiis-Evensen E. Phospholipase C isozymes are deregulated in colorectal cancer–insights gained from gene set enrichment analysis of the transcriptome. PloS One (2011) 6(9):e24419. doi: 10.1371/journal.pone.0024419 21909432 PMC3164721

[30] Chen D-T Hernandez JM Shibata D McCarthy SM Humphries LA Clark W. Complementary strand microRNAs mediate acquisition of metastatic potential in colonic adenocarcinoma. J Gastrointestinal Surg (2012) 16(5):905–13. doi: 10.1007/s11605-011-1815-0 PMC6753785 22362069

[31] Chauvin A Bergeron D Vencic J Lévesque D Paquette B Scott MS. Downregulation of KRAB zinc finger proteins in 5-fluorouracil resistant colorectal cancer cells. BMC Cancer (2022) 22(1):363. doi: 10.1186/s12885-022-09417-3 35379199 PMC8981854

[32] Ubink I Bloemendal HJ Elias SG Brink MA Schwartz MP Holierhoek YCW. Imatinib treatment of poor prognosis mesenchymal-type primary colon cancer: a proof-of-concept study in the preoperative window period (ImPACCT). BMC Cancer (2017) 17(1):282. doi: 10.1186/s12885-017-3264-y 28424071 PMC5395860

[33] Pusztai L Yau C Wolf DM Han HS Du L Wallace AM. Durvalumab with olaparib and paclitaxel for high CCCs score HER2-negative stage II/III breast cancer: Results from the adaptively randomized I-SPY2 trial. Cancer Cell (2021) 39(7):989–998.e5. doi: 10.1016/j.ccell.2021.05.009 34143979 PMC11064785

[34] Hatzis C Pusztai L Valero V Booser DJ Esserman L Lluch A. A genomic predictor of response and survival following taxane-anthracycline chemotherapy for invasive breast cancer. Jama (2011) 305(18):1873–81. doi: 10.1001/jama.2011.593 PMC5638042 21558518

[35] Itoh M Iwamoto T Matsuoka J Nogami T Motoki T Shien T. Estrogen receptor (ER) mRNA expression and molecular subtype distribution in ER-negative/progesterone receptor-positive breast cancers. Breast Cancer Res Treat (2014) 143(2):403–9. doi: 10.1007/s10549-013-2763-z 24337596

[36] Baldasici O Balacescu L Cruceriu D Roman A Lisencu C Fetica B. Circulating small EVs miRNAs as predictors of pathological response to neo-adjuvant therapy in breast cancer patients. Int J Mol Sci (2022) 23(20):1–20. doi: 10.3390/ijms232012625 PMC9604084 36293478

[37] Horak CE Pusztai L Xing G Trifan OC Saura C Tseng L-M. Biomarker analysis of neoadjuvant doxorubicin/cyclophosphamide followed by ixabepilone or paclitaxel in early-stage breast cancer. Clin Cancer Res (2013) 19(6):1587–95. doi: 10.1158/1078-0432.CCR-12-1359 23340299

[38] Wolf DM Yau C Wulfkuhle J Brown-Swigart L Gallagher RI Lee PRE. Redefining breast cancer subtypes to guide treatment prioritization and maximize response: Predictive biomarkers across 10 cancer therapies. Cancer Cell (2022) 40(6):609–623.e6. doi: 10.1016/j.ccell.2022.05.005 35623341 PMC9426306

[39] Choi W Porten S Kim S Willis D Plimack ER Hoffman-Censits J. Identification of distinct basal and luminal subtypes of muscle-invasive bladder cancer with different sensitivities to frontline chemotherapy. Cancer Cell (2014) 25(2):152–65. doi: 10.1016/j.ccr.2014.01.009 PMC4011497 24525232

[40] Consortium TGO. The Gene Ontology resource: enriching a GOld mine. . Nucleic Acids Res (2020) 49(D1):D325–34. doi: 10.1093/nar/gkaa1113 PMC7779012 33290552

[41] Kawaguchi Y Kopetz S Newhook TE De Bellis M Chun YS. RAS, TP53Mutation status of , and is superior to mutation status of alone for predicting prognosis after resection of colorectal liver metastases. Clin Cancer Res (2019) 25(19):5843–51. doi: 10.1158/1078-0432.CCR-19-0863 PMC6774854 31221662

[42] Ogata H Goto S Sato K Fujibuchi W Bono H Kanehisa M. KEGG: kyoto encyclopedia of genes and genomes. Nucleic Acids Res (1999) 27(1):29–34. doi: 10.1093/nar/27.1.29 9847135 PMC148090

[43] Yoshihara K Shahmoradgoli M Martínez E Vegesna R Kim H Torres-Garcia W. Inferring tumour purity and stromal and immune cell admixture from expression data. Nat Commun (2013) 4(1):2612. doi: 10.1038/ncomms3612 24113773 PMC3826632

[44] Chen B Khodadoust MS Liu CL Newman AM Alizadeh AA. Profiling tumor infiltrating immune cells with CIBERSORT. Methods Mol Biol (Clifton N.J.) (2018) 1711:243–59. doi: 10.1038/ncomms3612 PMC5895181 29344893

[45] Charoentong P Finotello F Angelova M Rieder D Hackl H Trajanoski Z. Pan-cancer immunogenomic analyses reveal genotype-immunophenotype relationships and predictors of response to checkpoint blockade. Cell Rep (2017) 18(1):248–62. doi: 10.1016/j.celrep.2016.12.019 28052254

[46] Chen DS Mellman I. Oncology meets immunology: the cancer-immunity cycle. Immunity (2013) 39(1):1–10. doi: 10.1016/j.immuni.2013.07.012 23890059

[47] Xu L Deng C Pang B Zhang X Liu W Liao G. TIP: A web server for resolving tumor immunophenotype profiling. Cancer Res (2018) 78(23):6575–80. doi: 10.1158/0008-5472.CAN-18-0689 30154154

[48] Auslander N Zhang G Lee JS Frederick DT Miao B Moll T. Robust prediction of response to immune checkpoint blockade therapy in metastatic melanoma. Nat Med (2018) 24(10):1545–9. doi: 10.1038/s41591-018-0157-9 PMC6693632 30127394

[49] Kuipers E Grady WM Lieberman D Seufferlein T Sung JJ Boelens PG. Colorectal cancer. Nat Rev Dis Primers (2015) 1:15065. doi: 10.1038/nrdp.2015.65 27189416 PMC4874655

[50] Diaz Z Aguilar-Mahecha A Paquet ER Basik M Orain M Camlioglu E. Next-generation biobanking of metastases to enable multidimensional molecular profiling in personalized medicine. Modern Pathol (2013) 26(11):1413–24. doi: 10.1038/modpathol.2013.81 23743930

[51] Gerovska D Larrinaga G Solano-Iturri JD Márquez J Gallastegi PG Khatib A-M. BRCA1An integrative omics approach reveals involvement of in hepatic metastatic progression of colorectal cancer. Cancers (2020) 12(9):1413–24. doi: 10.3390/cancers12092380 PMC7565528 32842712

[52] Choi EJ Yoo NJ Kim MS An CH Lee SH. Putative tumor suppressor genes EGR1 and BRSK1 are mutated in gastric and colorectal cancers. Oncology (2016) 91(5):289–94. doi: 10.1159/000450616 27677186

[53] Lu Y Zhou X Liu Z Wang W Li F Fu W. Characteristic analysis of featured genes associated with stemness indices in colorectal cancer. Front Mol Biosci (2020) 7:563922. doi: 10.3389/fmolb.2020.563922 33134313 PMC7576097

[54] Fang Y Yu H Liang X Xu J Cai X. Chk1-induced CCNB1 overexpression promotes cell proliferation and tumor growth in human colorectal cancer. Cancer Biol Ther (2014) 15(9):1268–79. doi: 10.4161/cbt.29691 PMC4128869 24971465

[55] Zhao S Hao C-l Zhao E-h Jiang H-m Zheng H-c. The suppressing effects of dkk3 expression on aggressiveness and tumorigenesis of colorectal cancer. Front Oncol (2020) 10:600322. doi: 10.3389/fonc.2020.600322 33425757 PMC7794014

[56] Pavlič A Urh K Štajer K Boštjančič E Zidar N. Epithelial-mesenchymal transition in colorectal carcinoma: comparison between primary tumor, lymph node and liver metastases. Front Oncol (2021) 11:662806. doi: 10.3389/fonc.2021.662806 34046357 PMC8144630

[57] Momma T Okayama H Saitou M Sugeno H Yoshimoto N Takebayashi Y. Expression of circadian clock genes in human colorectal adenoma and carcinoma. Oncol Lett (2017) 14(5):5319–25. doi: 10.3892/ol.2017.6876 PMC5661361 29113166

[58] Mittal S Aslam A Doidge R Medica R Winkler GS. The Ccr4a (CNOT6) and Ccr4b (CNOT6L) deadenylase subunits of the human Ccr4-Not complex contribute to the prevention of cell death and senescence. Mol Biol Cell (2011) 22(6):748–58. doi: 10.1091/mbc.e10-11-0898 PMC3057700 21233283

[59] Flanagan J Healey S Young J Whitehall V Chenevix-Trench G. Analysis of the transcription regulator, CNOT7, as a candidate chromosome 8 tumor suppressor gene in colorectal cancer. Int J Cancer (2003) 106(4):505–9. doi: 10.1002/ijc.11264 12845644

[60] Ding X Duan H Luo H. Identification of core gene expression signature and key pathways in colorectal cancer. Front Genet (2020) 11:45. doi: 10.3389/fgene.2020.00045 32153633 PMC7046836

[61] Shibata E Dutta A. A human cancer cell line initiates DNA replication normally in the absence of ORC5 and ORC2 proteins. J Biol Chem (2020) 295(50):16949–59. doi: 10.1074/jbc.RA120.015450 PMC7863895 32989049

[62] Raab M Sanhaji M Matthess Y Hörlin A Lorenz I Dötsch C. PLK1 has tumor-suppressive potential in APC-truncated colon cancer cells. Nat Commun (2018) 9(1):1106. doi: 10.1038/s41467-018-03494-4 29549256 PMC5856809

[63] Moon S Son HJ Mo HY Choi EJ Yoo NJ Lee SH. Mutation and expression alterations of histone methylation-related NSD2, KDM2B and SETMAR genes in colon cancers. Pathology Res Pract (2021) 219:153354. doi: 10.1016/j.prp.2021.153354 33621919

[64] Escudero-Paniagua B Bartolomé RA Rodríguez S Los Ríos VD Pintado L Jaén M. PAUF/ZG16B promotes colorectal cancer progression through alterations of the mitotic functions and the Wnt/β-catenin pathway. Carcinogenesis (2020) 41(2):203–13. doi: 10.1093/carcin/bgz093 31095674

[65] Chen J. The cell-cycle arrest and apoptotic functions of p53 in tumor initiation and progression. Cold Spring Harbor Perspect Med (2016) 6(3):a026104. doi: 10.1101/cshperspect.a026104 PMC4772082 26931810

[66] Zasadil LM Britigan EMC Ryan SD Kaur C Guckenberger DJ Beebe DJ. High rates of chromosome missegregation suppress tumor progression but do not inhibit tumor initiation. Mol Biol Cell (2016) 27(13):1981–9. doi: 10.1091/mbc.E15-10-0747 PMC4927272 27146113

[67] Silk A Zasadil LM Holland AJ Vitre B Cleveland DW Weaver BA. Chromosome missegregation rate predicts whether aneuploidy will promote or suppress tumors. Proc Natl Acad Sci United States America (2013) 110(44):E4134–41. doi: 10.1073/pnas.1317042110 PMC3816416 24133140

[68] Birkbak N Eklund AC Li Q McClelland SE Endesfelder D Tan P. Paradoxical relationship between chromosomal instability and survival outcome in cancer. Cancer Res (2011) 71(10):3447–52. doi: 10.1158/0008-5472.CAN-10-3667 PMC3096721 21270108

[69] Cancer Genome Atlas Network. Comprehensive molecular characterization of human colon and rectal cancer. Nature (2012) 487(7407):330–7. doi: 10.1158/0008-5472.can-10-366 PMC3401966 22810696

[70] Schell MJ Yang M Teer JK Lo FY Madan A Coppola D. A multigene mutation classification of 468 colorectal cancers reveals a prognostic role for APC. Nat Commun (2016) 7:11743. doi: 10.1038/ncomms11743 27302369 PMC4912618

[71] Gmeiner W Hellmann G Shen P. Tissue-dependent and -independent gene expression changes in metastatic colon cancer. Oncol Rep (2008) 19(1):245–51. doi: 10.3892/or.19.1.245 18097602

[72] Long L Yang W Liu L Tobias DK Katagiri R Wu K. Dietary intake of branched-chain amino acids and survival after colorectal cancer diagnosis. Int J Cancer (2020) 19:245–51. doi: 10.1002/ijc.33449 PMC8213867 33341092

[73] Zhang H-L Zhang A-H Zhou X-H Sun H Wang X-Q Liang L. High-throughput lipidomics reveal mirabilite regulating lipid metabolism as anticancer therapeutics. RSC Adv (2018) 8(62):35600–10. doi: 10.1039/C8RA06190D PMC9087915 35547938

[74] Chen X Shi B-L Qi R-Z Chang X Zheng H-G. Ultra-performance liquid chromatography/mass spectrometry-based metabolomics for discovering potential biomarkers and metabolic pathways of colorectal cancer in mouse model (ApcMin/+) and revealing the effect of honokiol. Front Oncol (2021) 11(3544). doi: 10.3389/fonc.2021.671014 PMC8473824 34589420

[75] Bond A Greenwood R Lewis S Corfe B Sarkar S O'Toole P. Volatile organic compounds emitted from faeces as a biomarker for colorectal cancer. Alimentary Pharmacol Ther (2019) 49(8):1005–12. doi: 10.1111/apt.15140 PMC6593415 30828825

[76] Hu J Yu A Othmane B Qiu D Li H Li C. Siglec15 shapes a non-inflamed tumor microenvironment and predicts the molecular subtype in bladder cancer. Theranostics (2021) 11(7):3089–108. doi: 10.7150/thno.53649 PMC7847675 33537076

[77] Gordon-Alonso M Hirsch T Wildmann C Bruggen Pvd. Galectin-3 captures interferon-gamma in the tumor matrix reducing chemokine gradient production and T-cell tumor infiltration. Nat Commun (2017) 8(1):793. doi: 10.1038/s41467-017-00925-6 28986561 PMC5630615

[78] Li J Ybarra R Mak J Herault A De Almeida P Arrazate A. IFNγ-induced chemokines are required for CXCR3-mediated T-cell recruitment and antitumor efficacy of anti-HER2/CD3 bispecific antibody. Clin Cancer Res (2018) 24(24):6447–58. doi: 10.1158/1078-0432.CCR-18-1139 29950350

[79] Workel HH Lubbers JM Arnold R Prins TM Vlies Pvd de Lange K. A transcriptionally distinct CXCL13+CD103+CD8+ T-cell population is associated with B-cell recruitment and neoantigen load in human cancer. Cancer Immunol Res (2019) 7(5):784–96. doi: 10.1158/2326-6066.CIR-18-0517 30872264

[80] Mueller L Goumas FA Himpel S Brilloff S Rogiers X Broering DC. Imatinib mesylate inhibits proliferation and modulates cytokine expression of human cancer-associated stromal fibroblasts from colorectal metastases. Cancer Lett (2007) 250(2):329–38. doi: 10.1016/j.canlet.2006.10.024 17141949

