# Supplementary material for: Transcriptomic correlates of cell cycle checkpoints with distinct prognosis, molecular characteristics, immunological regulation, and therapeutic response in colorectal adenocarcinoma
Source: Front Immunol. 2023 Dec 8;14:1291859. doi: 10.3389/fimmu.2023.1291859 (PMC10749195; doi:10.3389/fimmu.2023.1291859)
Supplement: Supplementary file 1 [file Table_1.docx]

**Table S1.** Hazard ratios and coefficients of CCCs involved in signature

| **Gene** | **Coefficient** | **p-value** |
| --- | --- | --- |
| *BRCA1* | -0.0245 | 0.0021 |
| *BRSK1* | 0.3478 | 0.0006 |
| *BUB1* | 0.2952 | 0.0073 |
| *CCNB1* | 0.4072 | 0.0004 |
| *CDC25C* | -0.5616 | 0.0001 |
| *CDK5RAP2* | 0.6340 | 0.0002 |
| *CDKN1B* | -0.2152 | 0.0001 |
| *CLOCK* | -0.2020 | 0.0005 |
| *CNOT6* | -0.0678 | 0.0123 |
| *CNOT6L* | -0.3124 | 0.0007 |
| *CNOT7* | -0.3678 | 0.0010 |
| *MAD1L1* | -0.1327 | 0.0014 |
| *MAD2L1* | 0.0293 | 0.0053 |
| *NBN* | -0.0601 | 0.0070 |
| *ORC1* | -0.1906 | 0.0006 |
| *PLK1* | -0.1149 | 0.0050 |
| *RGCC* | 0.1927 | 0.0018 |
| *SETMAR* | -0.4629 | 0.0458 |
| *ZNF207* | 0.1316 | 0.0055 |
| *ZW10* | 0.2037 | 0.0083 |

CCCs: Cell cycle checkpoints; HR: Hazard ratio; CI: Confidence interval
