# Supplementary figures and images for: Transcriptomic correlates of cell cycle checkpoints with distinct prognosis, molecular characteristics, immunological regulation, and therapeutic response in colorectal adenocarcinoma
Source: Front Immunol. 2023 Dec 8;14:1291859. doi: 10.3389/fimmu.2023.1291859 (PMC10749195; doi:10.3389/fimmu.2023.1291859)

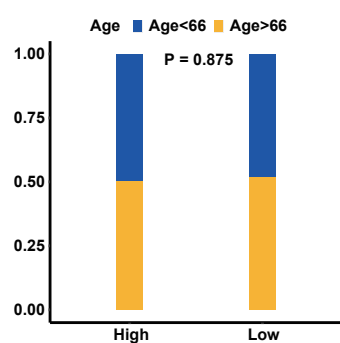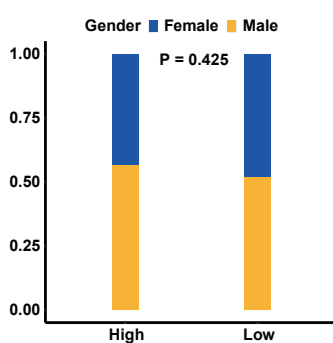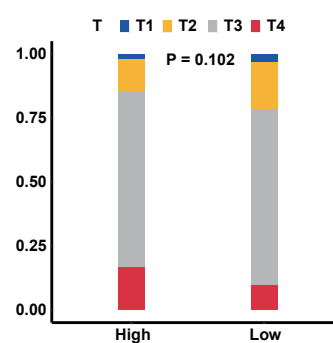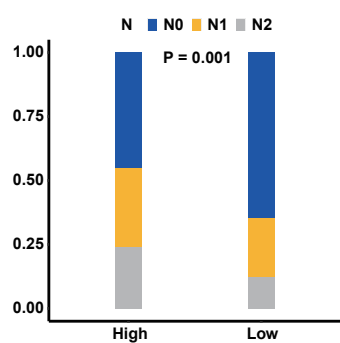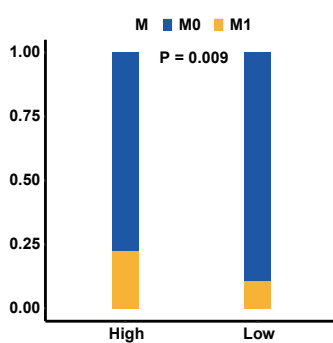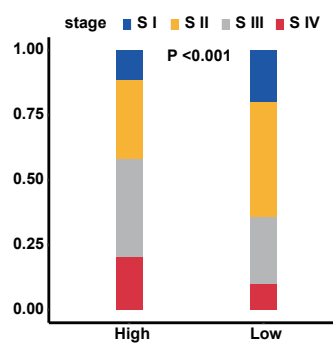

Supplement: Supplementary file 2 [file Image_1.pdf]

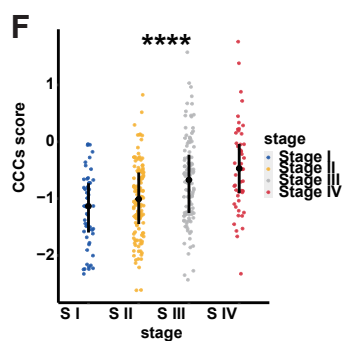

Supplement: Supplementary file 3 [file Image_2.pdf]

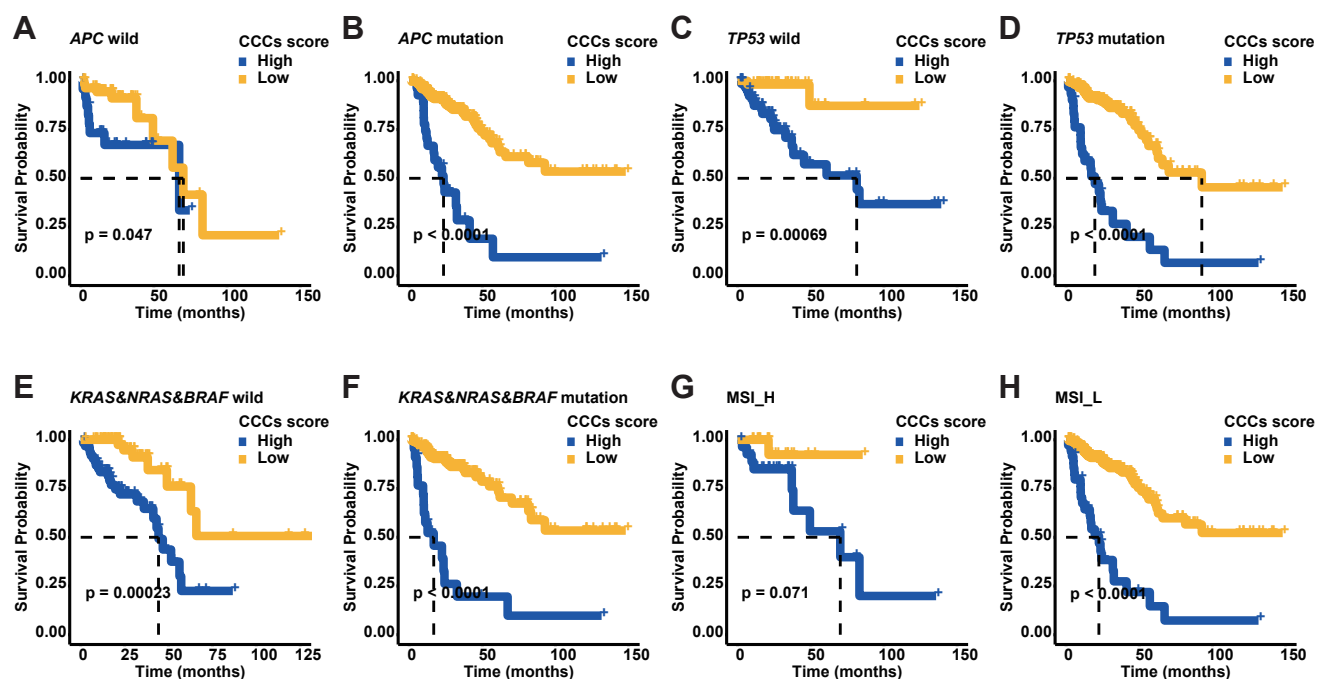

Supplement: Supplementary file 4 [file Image_3.pdf]

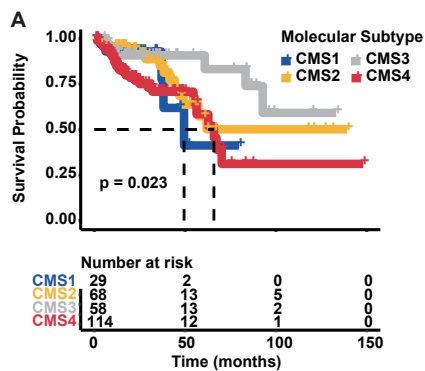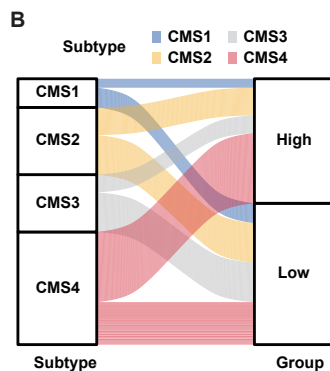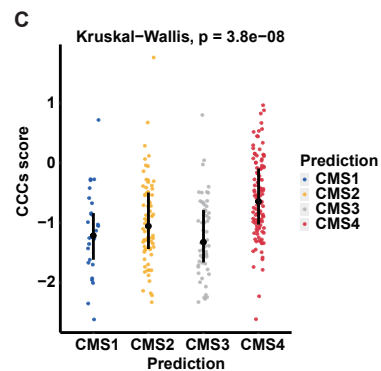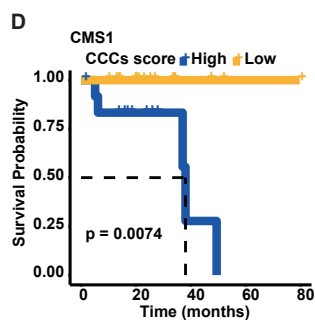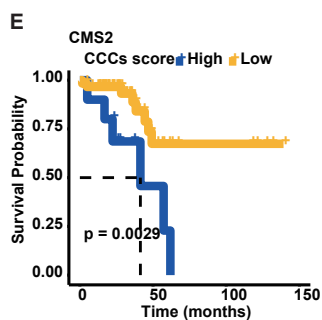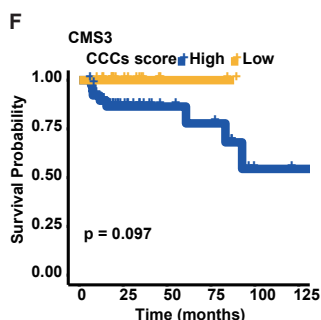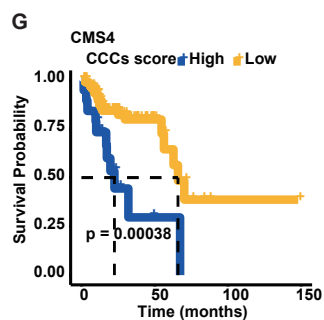

Supplement: Supplementary file 5 [file Image_4.pdf]

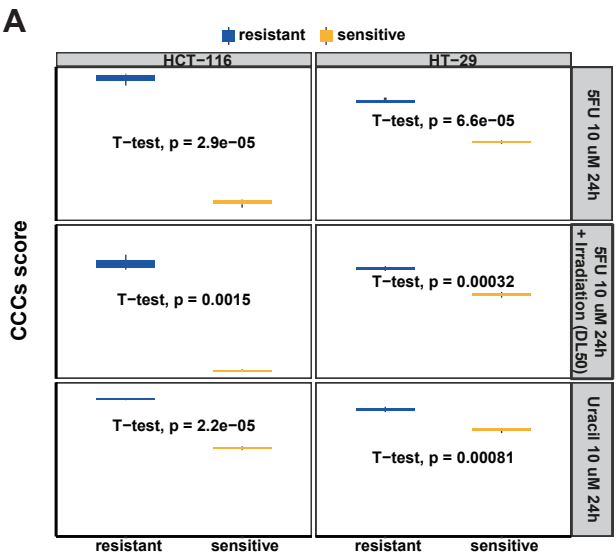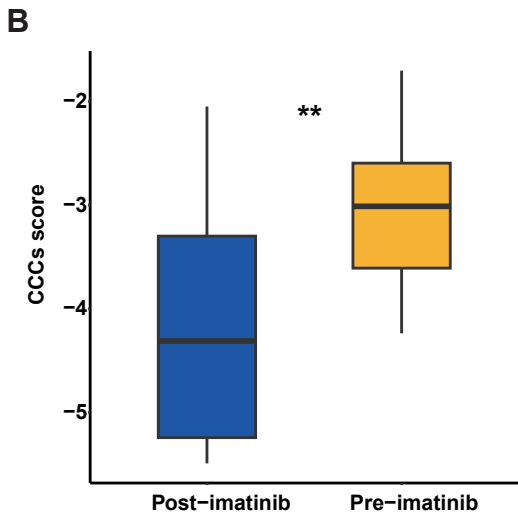

Supplement: Supplementary file 6 [file Image_5.pdf]
